# Genomic and phenotypic insights into the ecology of *Arthrobacter* from Antarctic soils

**DOI:** 10.1186/s12864-015-1220-2

**Published:** 2015-02-05

**Authors:** Melissa Dsouza, Michael W Taylor, Susan J Turner, Jackie Aislabie

**Affiliations:** Centre for Microbial Innovation, School of Biological Sciences, University of Auckland, Private Bag 92019, Auckland, 1142 New Zealand; BioDiscovery New Zealand Limited, Parnell, Auckland New Zealand; Landcare Research, Private Bag 3127, Hamilton, New Zealand

**Keywords:** Antarctica, Comparative genomics, *Arthrobacter*

## Abstract

**Background:**

Members of the bacterial genus *Arthrobacter* are both readily cultured and commonly identified in Antarctic soil communities. Currently, relatively little is known about the physiological traits that allow these bacteria to survive in the harsh Antarctic soil environment. The aim of this study is to investigate if Antarctic strains of *Arthrobacter* owe their resilience to substantial genomic changes compared to *Arthrobacter* spp. isolated from temperate soil environments.

**Results:**

Quantitative PCR-based analysis revealed that up to 4% of the soil bacterial communities were comprised of *Arthrobacter* spp. at four locations in the Ross Sea Region. Genome analysis of the seven Antarctic *Arthrobacter* isolates revealed several features that are commonly observed in psychrophilic/psychrotolerant bacteria. These include genes primarily associated with sigma factors, signal transduction pathways, the carotenoid biosynthesis pathway and genes induced by cold-shock, oxidative and osmotic stresses. However, these genes were also identified in genomes of seven temperate *Arthrobacter* spp., suggesting that these mechanisms are beneficial for growth and survival in a range of soil environments. Phenotypic characterisation revealed that Antarctic *Arthrobacter* isolates demonstrate significantly lower metabolic versatility and a narrower salinity tolerance range compared to temperate *Arthrobacter* species. Comparative analyses also revealed fewer protein-coding sequences and a significant decrease in genes associated with transcription and carbohydrate transport and metabolism in four of the seven Antarctic *Arthrobacter* isolates. Notwithstanding genome incompleteness, these differences together with the decreased metabolic versatility are indicative of genome content scaling.

**Conclusions:**

The genomes of the seven Antarctic *Arthrobacter* isolates contained several features that may be beneficial for growth and survival in the Antarctic soil environment, although these features were not unique to the Antarctic isolates. These genome sequences allow further investigations into the expression of physiological traits that enable survival under extreme conditions and, more importantly, into the ability of these bacteria to respond to future perturbations including climate change and human impacts.

**Electronic supplementary material:**

The online version of this article (doi:10.1186/s12864-015-1220-2) contains supplementary material, which is available to authorized users.

## Background

Antarctica is largely covered by glacial ice sheets, with ice-free areas making up _~_0.32% of the entire continental land mass [1]. Of these ice-free areas, 90% are located along the continental coastline and occur on the Antarctic Peninsula and the Ross Sea Region (RSR). Soils of the RSR are exposed to a wide range of environmental extremes including physical extremes of temperature and elevated ultraviolet (UV) radiation, as well as geochemical extremes of high salinity, low water and low nutrient availability [2]. Together these environmental conditions make Antarctic soils some of the harshest environments on Earth.

Prior to the advent of molecular ecology techniques, cultivation- and microscopy-based studies had reported that Antarctic soils are dominated by a few, cosmopolitan groups of bacteria [3,4]. However, modern molecular methods have allowed for a more accurate assessment of bacterial community composition. Pyrosequencing and clone libraries of the 16S rRNA gene from RSR soils have identified representatives of 15 phyla including *Acidobacteria*, *Actinobacteria*, *Armatimonadetes*, *Bacteroidetes*, *Chloroflexi*, *Cyanobacteria*, *Deinococcus*-*Thermus*, *Firmicutes*, *Gemmatimonadetes*, *Nitrospira*, *Planctomycetes*, *Proteobacteria*, *Spirochaetes*, *Verrucomicrobia* and Candidate ‘TM7’ [5-13].

While modern techniques have aided our understanding of bacterial diversity in Antarctic terrestrial environments, they have also revealed adaptive mechanisms of psychrophilic organisms through genomic data. At the time of writing, genomes of 46 psychrophilic/psychrotolerant bacteria and archaea were complete and published (as reviewed by De Maayer *et al*. [14]). Of these, just four studies have investigated psychrophilic/psychrotolerant organisms isolated from Antarctic environments including *Methanococcoides burtonii* from Ace Lake, Vestfold Hills [15,16], *Exiguobacterium antarcticum* from microbial mats, Lake Fryxell [17], *Octadecabacter antarcticus* from Antarctic sea ice [18], and *Cellulophaga algicola* from the surface of a sea-ice diatom *Melosira*, East Antarctica [19]. These studies have reported the presence of cold adaptation relating to membrane modification, compatible solute accumulation, reactive oxygen species (ROS) detoxification, and significant changes in bacterial protein sequences including reduction in charged residues, hydrophobic clusters and proline content. These genomes have provided an insight into the lifestyle of psychrophilic microorganisms in permanently cold environments (<5°C) such as the Antarctic marine and lacustrine environments. However, there remains a gap in our knowledge of the adaptive mechanisms of psychrotolerant bacteria isolated from Antarctic terrestrial environments wherein large temperature fluctuations are common. In soils of the RSR, members of the *Acidobacteria*, *Actinobacteria*, *Bacteroidetes*, *Deinococcus*-*Thermus* and *Proteobacteria* phyla represent dominant taxa [5-7,9-12]. Of these, *Actinobacteria* are of special note. The phylum *Actinobacteria* is composed of phylogenetically diverse organisms that have been primarily investigated for their ability to cause disease in plants and animals, to produce anti-microbial compounds and anti-tumour agents, and to degrade recalcitrant molecules in soil environments [20]. Within *Actinobacteria*, members of the genus *Arthrobacter* are of note as they are among the most frequently isolated bacteria, occurring most commonly in soils and environments contaminated with industrial chemicals and radioactive materials. Their ubiquity can be attributed to their nutritional versatility and their resistance to environmental stressors [21]. At the time of writing, *Arthrobacter* included 82 species with validly published names (http://www.bacterio.net/a/). Of these, complete and published genomes are available for just six species, namely *A. aurescens* TC1, *A. chlorophenolicus* A6, *Arthrobacter* sp. FB24, *A. nitroguajacolicus* Rue61a, *A. phenanthrenivorans* Sphe3, and *A. arilaitensis* re117 [22].

In soils of the RSR, *Arthrobacter* species are both readily cultured and commonly observed in 16S rRNA gene clone libraries. Furthermore, they can be dominant in the soil environment as observed in soils on the Hatherton Drift, Transantarctic Mountains [5]. Despite their prevalence in soils of the RSR, very little is known about the physiological traits that allow these organisms to survive, flourish and establish dominance in the harsh Antarctic soil environment. A key question is if Antarctic strains of *Arthrobacter* owe their resilience to substantial genomic changes compared to *Arthrobacter* spp. isolated from temperate soil environments. Therefore, the three objectives of this study were (1) to investigate the abundance and diversity of *Arthrobacter* species found in soil microbial communities at four locations in the RSR, (2) to compare genomes of seven Antarctic *Arthrobacter* isolates with seven temperate *Arthrobacter* spp., focusing on traits that may contribute to survival and growth in the Antarctic soil environment, and (3) to investigate the metabolic versatility and salinity tolerance range of Antarctic *Arthrobacter* isolates compared to three temperate, soil-dwelling *Arthrobacter* species. For this, a combination of genotypic and phenotypic techniques including quantitative PCR (qPCR), whole genome sequencing and BIOLOG’s Phenotype Microarray (PM) technologies were utilised. To our knowledge this is the first study to provide genomic and phenotypic insights into the metabolic potential and ecological role of *Arthrobacter* strains isolated from RSR soils.

## Results and discussion

### Abundance and diversity of *Arthrobacter* spp. in soils of RSR

**Figure 1:**
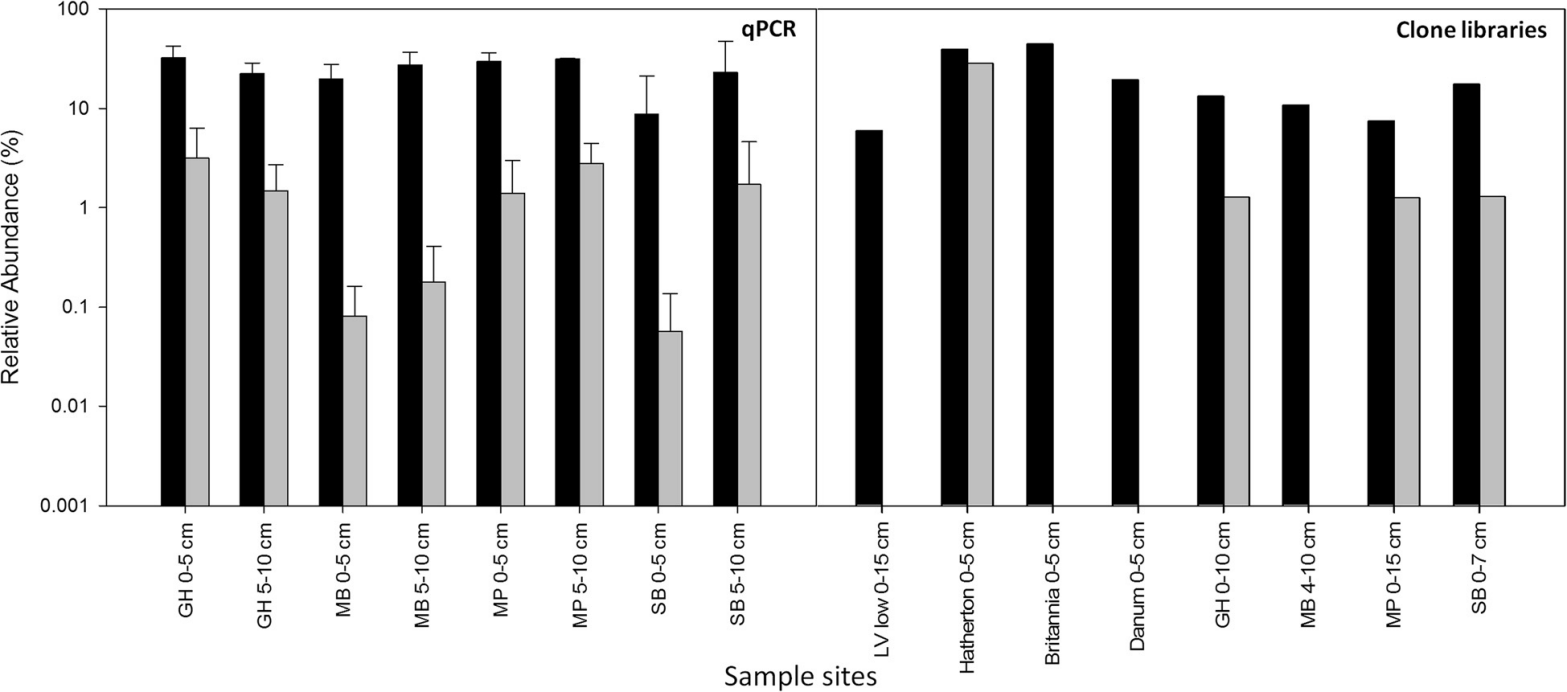
**Relative abundance of bacteria belonging to the phylum** ***Actinobacteria*** **and genus** ***Arthrobacter*** **in soils of RSR determined by 16S rRNA gene clone libraries [** 5 **-** 7 **,** 9 **,** 23 **] and by qPCR data (this study).** Black bar represents *Actinobacteria*, grey bar represents *Arthrobacter*. Bar represents standard deviation for qPCR data. Sample sites: SB, Scott Base; MP, Marble Point; MB, Minna Bluff; GH, Granite Harbour; LV, Luther Vale.

A total of eight samples from two soil depths at four locations within the RSR was investigated by qPCR (Figure 1) to determine the relative abundance of members of the phylum *Actinobacteria* and genus *Arthrobacter*. Specificity of the qPCR assays were tested by clone library preparations with DNA from three Antarctic soil samples and a marine sponge sample. All sequenced clones belonged to the correct target group. The relative abundance of each bacterial taxon was calculated as a ratio of measured copy numbers for each taxon-specific qPCR assay to measured copy numbers for the ‘all *Bacteria*’ assay. Members of *Actinobacteria* and *Arthrobacter* were present at all sample sites. *Actinobacteria* represented approximately 10-40% of the bacterial community at all soil locations, consistent with published data based on 16S rRNA gene clone libraries (as observed in Figure 1) [5-9,23]. In this study, up to 4% of the bacterial community was comprised of *Arthrobacter* species, with the lowest relative abundance observed in soils of Minna Bluff and the highest in soils of Granite Harbour. These results were also broadly consistent with published data (as observed in Figure 1) [5-9,23].

Phylogenetic analysis was performed on 16S rRNA gene sequences of clones and isolates associated with *Arthrobacter* from soils of the RSR. This analysis revealed that clones and isolates clustered together, clearly illustrating that the cultured isolates are representative of *Arthrobacter* spp. observed in soil bacterial communities (Additional file 1). Of these, seven isolates that represent the phylogenetic diversity of *Arthrobacter* spp. occurring in RSR soils were chosen for subsequent comparative analyses by whole genome sequencing and phenotypic characterisation.

### Genome analyses

#### Genome overview

**Figure 2:**
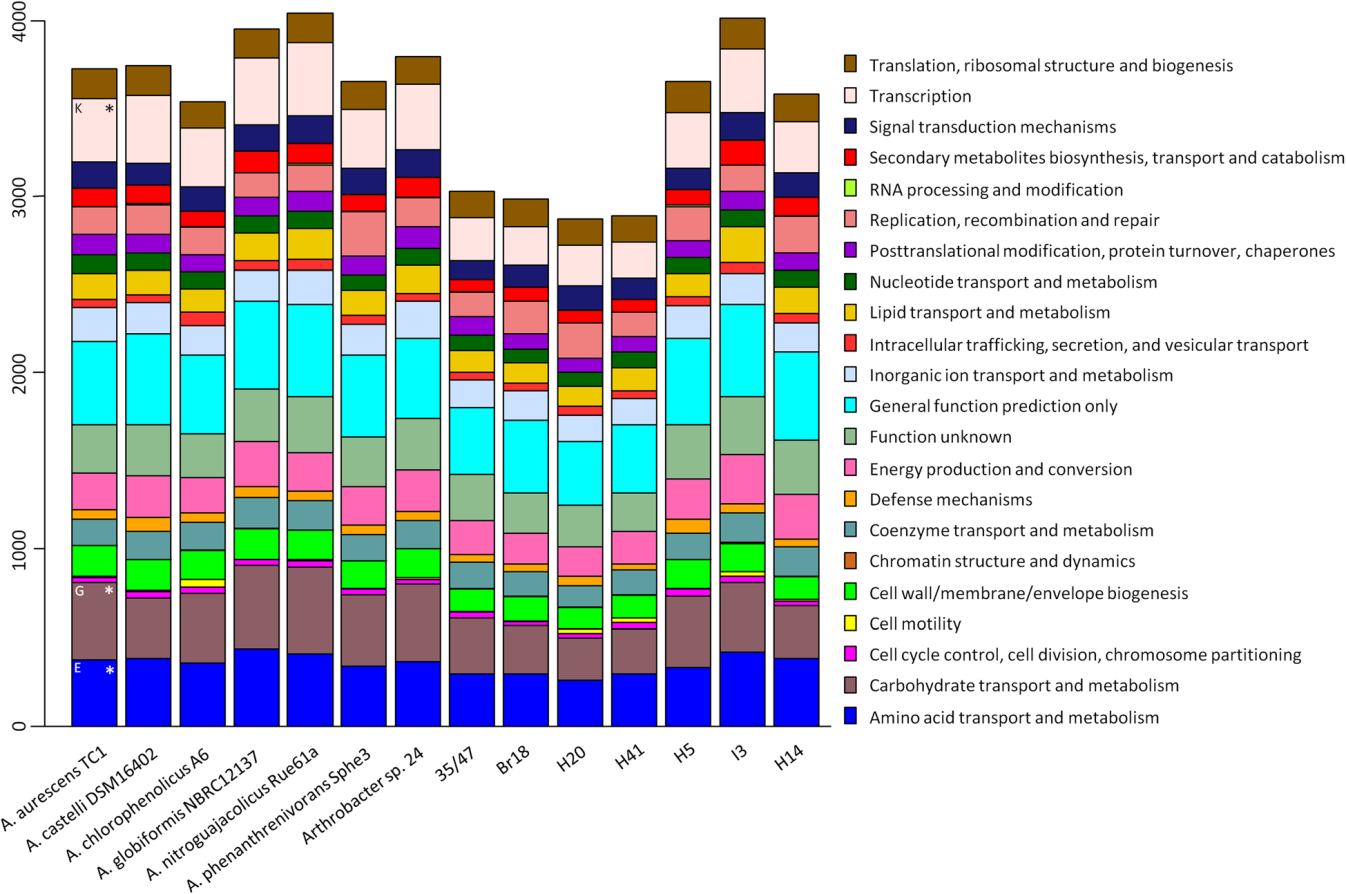
**Comparison of gene content in seven temperate** ***Arthrobacter*** **spp. and seven Antarctic** ***Arthrobacter*** **isolates by COG categories.** Asterisks represent abundant COG categories. Letters K, G, and E represent COG categories transcription, carbohydrate and amino acid transport and metabolism, respectively.

Genomes of the seven Antarctic *Arthrobacter* isolates are composed of the chromosome, each constructed from varying numbers of DNA scaffolds ranging from 53 to 158. The completeness of the seven genomes ranged from 78-98%, assessed by the occurrence of essential, single-copy genes. Due to this incompleteness, one must regard with caution the apparent absence or low copy number of a given gene. General genome features of the seven Antarctic *Arthrobacter* strains, as compared with the seven temperate *Arthrobacter* spp., are listed in Table 1. The genome G + C content for the seven Antarctic strains ranges from approx. 61-65%, similar to that for the seven temperate *Arthrobacter* species. The genomes contain 3,429-4,772 open reading frames (ORFs) with an average coding density of 88%. While genomes of strains I3, H5 and H14 contain 4,703, 4,552 and 4,566 protein-coding sequences (CDSs) respectively, significantly fewer CDSs were observed in genomes of strains H20 (3,466), 35/47 (3,470), Br18 (3,575) and H41 (3,373) (P < 0.01). For each genome, of the total CDSs, approx. 74% of the CDSs were classified into clusters of orthologous groups (COG) categories. Notably, the highest percentage of genes was assigned to COG categories such as amino acid transport and metabolism [E], carbohydrate transport and metabolism [G], and transcription [K] (Figure 2). Putative horizontally transferred genes, identified by the Joint Genome Institute (JGI) annotation pipeline, constituted 1.53-3.79% of the total genes observed. All Antarctic strains harboured a high number of genes associated with mobile genetic elements (up to 2.4% of total genes) encoding for phage integrases, transposases, and other phage elements (Additional file 2). Two putative phage sequences were identified in the genome of strain Br18, and genomes of strains H20 and H14 contained one putative phage sequence each (Additional file 3) [24]. No phage sequences were identified in the remaining four Antarctic strains. Significantly fewer copies of the 16S rRNA gene were identified in Antarctic *Arthrobacter* genomes as compared to the temperate *Arthrobacter* spp. (P <0.05).

**Table 1:** **General genome features of seven Antarctic** ***Arthrobacter*** **strains (this study) vs. seven temperate** ***Arthrobacter*** **spp.** [22]

	**1**	**2**	**3**	**4**	**5**	**6**	**7**	**8**	**9**	**10**	**11**	**12**	**13**	**14**
**Genome data**														
Genome size (bp)	5,226,648	4,585,800	4,980,870	4,954,410	5,081,038	4,535,320	5,070,478	3,554,370	3,562,925	4,340,329	3,452,356	3,321,099	4,379,674	4,835,497
DNA coding region (%)	4,602,246 (88.05)	4,096,643 (89.33)	4,458,001 (89.50)	4,420,17 (89.23)	4,572,987 (90)	4,024,613 (88.74)	4,552,065 (89.78)	3,169,499 (89.17)	3,140,572 (88.15)	3,765,602 (86.76)	3,090,905 (89.53)	2,903,224 (87.42)	3,887,579 (88.76)	4,164,528 (86.12)
G + C content (%)	62.43	63.58	65.96	66.21	62.25	65.37	65.39	63.76	65.28	60.57	62.30	63.98	61.29	65.18
Scaffold count	3	49	3	125	3	3	4	53	120	158	88	148	104	124
Total RNA genes	94	64	103	53	71	64	86	58	68	67	63	56	64	69
tRNA genes	54	46	88	50	53	50	51	43	51	48	44	44	49	53
16S rRNA genes	6	2	5	1	6	4	5	2	1	3	2	1	2	2
Other RNA genes	20	10	-	-	-	2	20	9	11	12	12	9	9	9
Total number of genes	4,793	4,516	4,744	4,592	4,655	4,273	4,622	3,528	3,643	4,633	3,529	3,429	4,616	4,772
Total protein CDSs (%)	4699 (98.04)	4452 (98.58)	4641 (97.83)	452 (98.84)	4584 (98.47)	4209 (98.5)	4536 (98.14)	3470 (98.36)	3575 (98.13)	4566 (98.55)	3466 (98.21)	3373 (98.37)	4552 (98.61)	4703 (98.55)
With function prediction (%)	3419 (71.33)	3429 (75.93)	3125 (65.87)	2784 (60.76)	3800 (81.63)	3101 (72.57)	3279 (70.94)	2793 (79.17)	2750 (75.49)	3320 (71.66)	2660 (75.38)	2614 (76.23)	3393 (73.51)	3630 (76.07)
Without function prediction (%)	1280 (26.71)	1023 (22.65)	1516 (31.96)	1745 (38.08)	784 (18.64)	1108 (25.93)	1257 (27.20)	677 (19.19)	825 (22.65)	1246 (26.89)	806 (22.84)	759 (22.13)	1159 (25.11)	1073 (22.49)
With COGs (%)	3307 (69)	3339 (73.94)	3124 (65.84)	3546 (77.39)	3625 (77.87)	3297 (77.16)	3361 (72.72)	2743 (77.75)	2697 (74.03)	3221 (69.52)	2588 (73.34)	2584 (75.36)	3307 (71.64)	3591 (75.25)
Horizontally transferred genes (%)	242 (5.05)	188 (4.16)	239 (5.04)	83 (1.81)	-	68 (1.59)	149 (3.22)	73 (2.07)	97 (2.66)	129 (2.78)	117 (3.32)	74 (2.16)	175 (3.79)	73 (1.53)
Genome Completeness (%)	-	-	-	-	-	-	-	92.30	98	94.23	90.38	77.88	97.12	98
**Metadata**														
Isolation source	South Dakota	Mural painting Austria	Fort Collins, Colorado	Geneva, New York	Ruetgerswerke AG, Germany	Perivleptos, Greece	Seymour, Indiana	Scott Base, Ross Sea Region	Britannia, Darwin Mountains	Hatherton, Darwin Mountains	Hatherton, Darwin Mountains	Hatherton, Darwin Mountains	Hatherton, Darwin Mountains	Isca, Darwin Mountains
Habitat	Atrazine enriched soil	Biofilm	Soil	Soil	Sludge	Soil	Soil	Soil	Soil	Soil	Soil	Soil	Soil	Soil
Colony colour	NR	Light yellow on NA**	Pearl grey on PC^#^	Cream on SA^$^	Yellow on NA^%^	Yellow-cream on LB^$$^	NR	Yellow	Pink/red	cream	Yellow	Pink/red	Cream	cream
Temperature range (°C)	30*	15-37**	3-37^#^	NR	4-37^%^	4-37^$$^	4-37^%%^	5-37	5-25^##^	5-25^##^	5-25^##^	5-25^##^	5-25^##^	5-25^##^

1, *A. aurescens* TC1; 2, *A. castelli* DSM 16402; 3, *A. chlorophenolicus* A6; 4, *A. globiformis* NBRC 12137; 5, *A. nitroguajacolicus* Rue61a; 6, *A. phenanthrenivorans* Sphe3; 7, *Arthrobacter* sp. FB24; 8, 35/47; 9, Br18; 10, H14; 11, H20; 12, H41; 13, H5; 14, I3.

For Antarctic isolates, colony colour observed following incubation at 15°C for 3–4 d on R2A agar.

*, Data from [25]; **, Data from [26]; #, Data from [27]; $, Data from [28]; %, Data from [29]; $$, Data from [30]; %%, Data from [31]; ##, Data from [5].

NA, Nutrient Agar; LA, Luria-Bertani Agar; P-C, Peptone-Carbohydrate Agar; SA, Standard Agar; NR, not reported.

#### General genome comparisons

**Figure 3:**
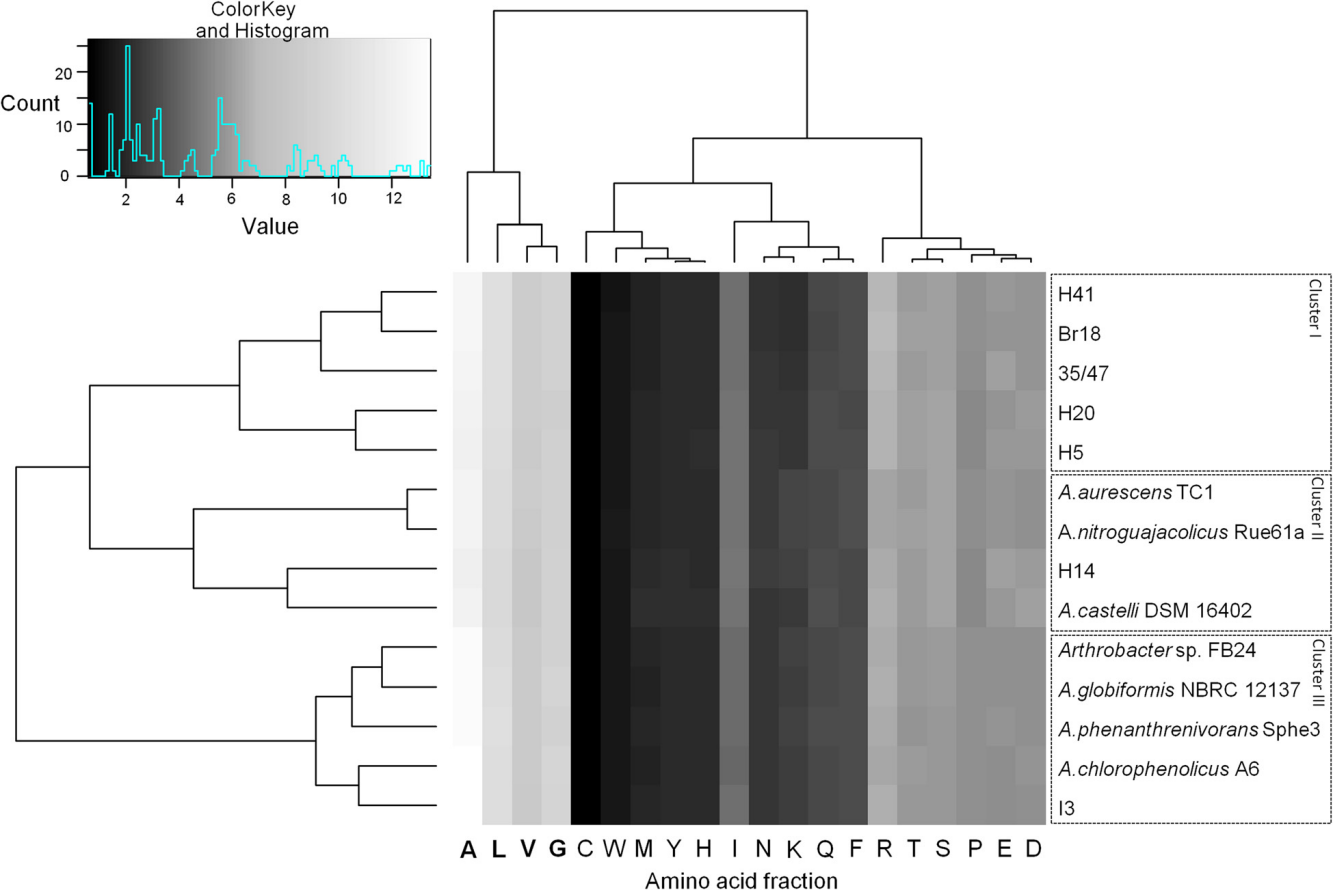
**Amino acid usage heatmap of seven temperate** ***Arthrobacter*** **spp. and seven Antarctic** ***Arthrobacter*** **isolates based on their protein content.** The percentage of amino acid usage was plotted in gplots using R. Amino acids highlighted in bold face represent abundant amino acids.

**Figure 4:**
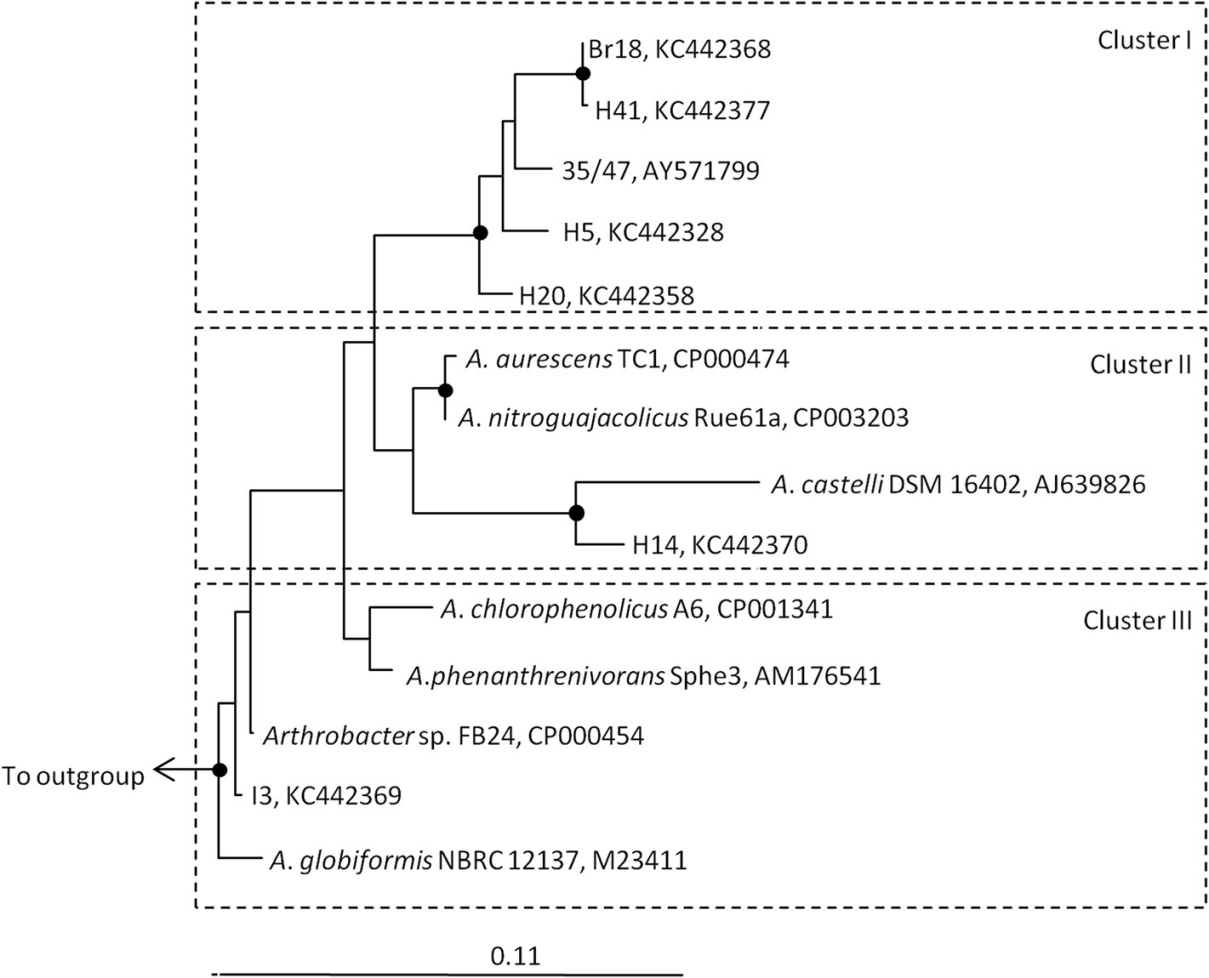
**Maximum likelihood phylogenetic tree based on 16S rRNA gene sequences from seven Antarctic** ***Arthrobacter*** **isolates and seven temperate** ***Arthrobacter*** **species.** Filled circles indicate bootstrap support of >90%, and open circles represent >75% support (maximum parsimony, 1000 resamplings). Bar, 0.11 substitutions per compared nucleotide site. Outgroup comprises *Microbacterium maritypicum*, AM 181506 and *M. profundi*, EF623999.

**Figure 5:**
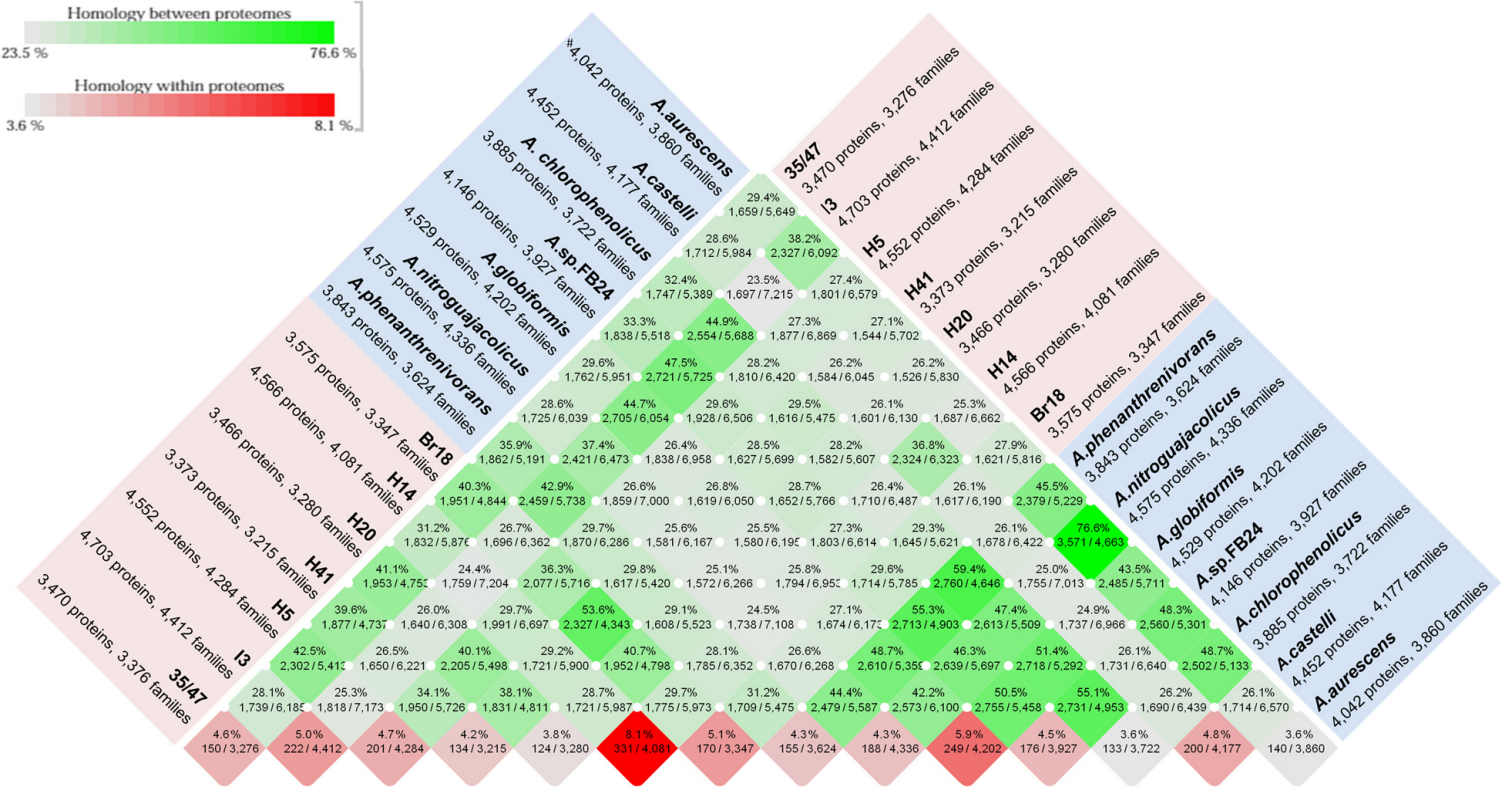
**BLAST matrix of an all against all protein comparison of 14** ***Arthrobacter*** **genomes.** The blue box contains genomes of seven temperate *Arthrobacter* spp. and the red box contains genomes of seven Antarctic *Arthrobacter* isolates. #, Note that proteins represent total CDSs and families represent CDSs not in paralogous clusters (unique CDSs). Red diamonds represent protein families in paralogous clusters.

General comparisons between genomes of seven temperate *Arthrobacter* spp. and seven Antarctic *Arthrobacter* strains were carried out using CMG-Biotools [32]. Firstly, amino acid usage was calculated for all 14 *Arthrobacter* isolates using their protein sequences. The amino acid usage tree (Figure 3) shows three main clusters. This clustering pattern is similar to the clustering observed in the maximum likelihood tree based on 16S rRNA gene sequences (Figure 4). This analysis revealed that Ala, Gly, Leu, and Val are the most frequently used amino acids across all *Arthrobacter* genomes. Predicted proteome comparisons and a pan- and core-genome plot analysis were also performed on all 14 *Arthrobacter* genomes using CMG-Biotools [32]. Proteomes were predicted for each isolate using Prodigal [33] and then BLAST algorithm (Basic Local Alignment Search Tool)-based proteome comparisons were performed to identify whether proteins are shared between predicted proteomes [34]. In Figure 5, the main part of the matrix (shaded green) consists of pairwise proteome comparisons and the bottom row (shaded red) represents a self-comparison where a hit within the proteome to a protein other than the query is identified as an internal homolog or a paralog. The BLAST matrix illustrates that conservation between Antarctic *Arthrobacter* genomes (24.4-53.6%) is low as compared to genomes of *Arthrobacter* spp. isolated from temperate soil environments (42.2-76.6%) (excluding *A. castelli* that was isolated from the biofilm of a mural). This observation was also supported by the pan- and core-genome plot analysis (Additional file 4), as the Antarctic core- and pan-genome comprised 1,285 and 10,873 gene families respectively and the temperate core- and pan-genome comprised 1,559 and 9,798 gene families respectively. Approximately 4.8% of the total CDSs were in paralogous clusters across all *Arthrobacter* genomes. The final *Arthrobacter* pan-genome comprised 14,902 sequences, indicative of a large diversity of accessory genes. The *Arthrobacter* core-genome is comprised of 1,153 gene families, representing approx. 27% of the total genes in *Arthrobacter* genomes. A large proportion of genes in the Antarctic and temperate core-genomes were assigned to COG categories including amino acid transport and metabolism [E], carbohydrate transport and metabolism [G] and translation, ribosomal structure and biogenesis [J].

### Genomic traits linked to environmental stress-related adaptation

Several known adaptive mechanisms for growth and survival in cold terrestrial environments were identified in the genomes of all 14 *Arthrobacter* isolates and are summarised in Table 2 (Additional file 5), and in the following categorical descriptions.

**Table 2:** **List of genes linked to environmental stress response**

**Product name**	**Gene symbol**	**Enzymes**	**COGs**	**1**	**2**	**3**	**4**	**5**	**6**	**7**	**8**	**9**	**10**	**11**	**12**	**13**	**14**
**SIGMA FACTORS**																	
DNA-directed RNA polymerase sigma 24	*rpoE*	-	COG1595	15	11	9	6	14	8	9	7	7	13	9	7	12	9
DNA-directed RNA polymerase sigma 28	*fliA*	-	COG1191	1	-	2	1	1	1	1	-	1	-	1	2	-	2
DNA-directed RNA polymerase, sigma 70	*rpoD*	-	COG0568	1	4	2	1	1	1	1	1	1	7	1	1	1	1
**OXIDATIVE STRESS RESPONSE**																	
Superoxide dismutase	*sodA*	EC:1.15.1.1	COG0605	1	1	1	1	2	1	1	1	1	1	1	1	1	1
Cu/Zn superoxide dismutase	*sodC*	EC:1.15.1.1	COG2032	-	-	-	-	-	-	-	-	-	-	1	-	-	-
Mn-containing catalase	-	-	COG3546	1	-	-	-	1	-	-	-	2	-	1	1	-	-
Catalase	*katE*	EC:1.11.1.6	COG0753	2	2	2	1	2	3	2	1	3	2	2	2	2	1
Catalase (peroxidase I)	*katG*	EC:1.11.1.21	COG0376	-	-	1	1	-	1	-	-	-	-	-	-	-	-
Peroxiredoxin	*bcp*	EC:1.11.1.15	COG1225	2	3	3	1	3	3	2	5	4	4	3	4	4	6
Organic hydroperoxide reductase	*osmC, ohr*	-	COG1764	3	2	3	3	3	3	3	2	2	2	3	2	2	3
Thioredoxin	*trx*	EC:1.8.1.8	COG3118	4	3	5	3	6	5	5	3	7	5	4	3	5	4
Thioredoxin reductase	*trxB*	EC:1.8.1.9	COG0492	4	4	3	3	3	6	2	4	5	4	1	4	2	2
Thiol-disulfide isomerase and thioredoxins	*trxA*	-	COG0526	2	3	2	-	1	2	5	4	3	2	2	1	3	1
Redox-sensitive transcriptional activator	*soxR*	-	COG0789	1	-	-	-	1	-	-	-	-	2	1	-	1	-
**OSMOPROTECTION**																	
***Glycogen metabolism***																	
Glycogen synthase	*glgA*	EC:2.4.1.21	COG0438	1	1	1	1	1	1	1	1	1	1	1	1	1	1
1,4-alpha-glucan branching enzyme	*glgB*	EC:2.4.1.18	COG0296	1	1	1	1	1	1	1	1	1	1	1	1	1	1
ADP-glucose pyrophosphorylase	*glgC*	EC:2.7.7.27	COG0448	1	1	1	1	1	1	1	2	2	1	1	2	1	1
Glucan phosphorylase	*glgP*	EC:2.4.1.1	COG0058	1	1	1	1	1	1	1	1	1	1	1	1	1	1
Glycogen debranching enzyme	*glgX*	EC:3.2.1.33	COG1523	2	2	2	3	2	1	2	2	2	2	2	2	2	2
***Trehalose metabolism***																	
Trehalose-6-phosphate synthase	*otsA*	EC:2.4.1.15	COG0380	1	1	1	1	1	1	1	1	1	1	1	1	1	1
Trehalose-6-phosphatase	*otsB*	EC:3.1.3.12	COG1877	2	1	1	1	1	1	1	1	1	1	1	1	1	1
Trehalose synthase	*treS*	EC:3.2.1.1, EC:5.4.99.16	COG0366	2	1	2	3	2	3	1	1	1	1	1	1	1	1
Maltooligosyl trehalose synthase	*treY*	EC:5.4.99.15	COG3280	1	1	1	1	1	1	1	1	1	1	1	1	1	1
Malto-oligosyltrehalose trehalohydrolase	*treZ*	EC:3.2.1.141	COG0296	1	1	1	1	1	1	1	1	1	1	1	1	1	1
***Glycine betaine/proline ABC transporter***																	
glycine betaine/proline ABC transporter - ATP binding subunit	*proV*	-	COG1125	2	3	1	1	3	1	1	2	3	3	2	3	3	1
glycine betaine/proline ABC transporter - membrane subunit	*proW*	-	COG1174	2	6	2	2	3	2	2	4	6	6	4	6	6	2
glycine betaine/proline ABC transporter - periplasmic binding protein	*proX*	-	COG1732	2	3	1	1	1	1	1	2	3	3	2	3	3	1
**COLD SHOCK RESPONSE**																	
Cold shock' DNA binding domain	*csd*	-	COG1278	3	4	5	4	3	4	4	3	3	4	3	3	2	4
CspA-like cold acclimation protein	*capA*	-	COG1278	1	1	1	1	1	1	1	1	-	1	-	-	1	1
Transcription elongation factor	*nusA*	-	COG0195	1	1	1	1	1	1	1	1	1	1	1	1	1	1
Polyribonucleotide nucleotidyltransferase	*pnp*	EC:2.7.7.8	COG1185	1	1	1	1	1	1	1	1	1	1	1	1	1	1
Ribosome-binding factor A	*rbfA*	-	COG0858	1	1	1	1	1	1	1	1	2	1	1	1	1	1
Translation initiation factor 1	*infA*	-	COG0361	1	1	1	1	1	1	1	1	1	1	-	1	1	1
Translation initiation factor 2	*infB*	-	COG0532	1	1	1	1	1	1	1	1	1	1	1	1	1	1
**MEMBRANE ADAPTATIONS**																	
Delta6-desaturase	*desA*	EC:1.14.19.3	COG3239	3	1	1	2	3	1	2	2	1	5	3	6	4	2
***Carotenoid biosynthesis***																	
Isopentenyldiphosphate δ isomerase	*idi*	EC 5.3.3.2	COG1443	1	1	-	-	1	-	-	1	1	1	1	1	-	-
Geranylgeranyl pyrophosphate synthase	*crtE*	EC:2.5.1.1, EC:2.5.1.10, EC:2.5.1.29	COG0142	1	2	-	-	1	1	-	2	2	1	2	4	2	1
Phytoene synthase	*crtB*	-	COG1562	1	1	-	-	1	-	-	1	1	1	1	1	-	-
Phytoene desaturase	*crtI*	EC:1.3.99.26, EC:1.3.99.28, EC:1.3.99.29, EC:1.3.99.31	COG1233	1	2	-	-	1	1	1	3	3	3	3	2	2	2
Lycopene elongase	*crtEB*	-	COG0382	1	1	-	-	1	-	-	1	1	1	1	1	-	-
Lycopene epsilon cyclase domain crtYe	*crtYe*	-	TIGR03462	1	1	-	-	-	-	-	1	-	1	1	-	-	-
Lycopene epsilon cyclase domain crtYf	*crtYf*	-	TIGR03462	-	1	-	-	-	-	-	1	-	1	1	-	-	-

1, *A. aurescens* TC1; 2, *A. castelli* DSM 16402; 3, *A. chlorophenolicus* A6; 4, *A. globiformis* NBRC 12137; 5, *A. nitroguajacolicus* Rue61a; 6, *A. phenanthrenivorans* Sphe3; 7, *Arthrobacter* sp. FB24; 8, 35/47; 9, Br18; 10, H14; 11, H20; 12, H41; 13, H5; 14, I3.

Numbers in each column represent copy numbers per genome. Locus tags for each copy number are listed in Additional file 5.

#### Sigma factors

Sigma factors are dissociable units of bacterial RNA polymerase that control the conditional expression of a specific set of genes in response to a particular stress or stimulus. Copies of genes of the σ^70^ factor, RpoD, more commonly referred to as the house-keeping or general stress response sigma factor, were identified in all Antarctic and temperate *Arthrobacter* genomes. The presence of multiple copies of genes for RpoD is a common feature in psychrophilic bacteria such as *Planococcus halocryophilus* [35] and *Psychromonas ingrahamii* [36]. Further analysis also revealed the presence of several copies of genes for the σ^24^ factor, RpoE, associated with regulating cellular responses to heat shock and other stresses on cell membrane and periplasmic proteins in all Antarctic and temperate *Arthrobacter* genomes. In *Escherichia coli*, RpoE also regulates cell lysis in a prolonged stationary phase, thus providing nutrients for the next generation of cells [37].

#### Oxidative stress response

Antarctic *Arthrobacter* genomes contain several copies of genes encoding (putative) oxidases that contribute to the abundance of endogenous H_2_O_2_ and other ROS (Additional file 6). Furthermore, ROS are formed at a higher abundance as a result of increased oxygen solubility at low temperatures [38]. Consequently, combating free radical damage alongside surviving exposure to UV radiation is crucial for survival in the Antarctic soil environment. Genomes of the Antarctic *Arthrobacter* isolates contain several genes that confer protection from free radical damage and allow for detoxification of ROS. This includes up to two copies of the superoxide dismutase gene, *sodA* and up to three copies of the catalase gene that were identified in all Antarctic *Arthrobacter* genomes. Additionally, several copies of the peroxiredoxin gene, *bcp* and thioredoxin genes, *trx*, *trxB* were identified in all Antarctic *Arthrobacter* genomes. Genes for antioxidant activity have also been identified in other cold-adapted bacteria including *Colwellia psychrerythraea* [39], *Desulfotalea psychrophila* [40], and *P. halocryophilus* [35]. These genes were identified in genomes of all temperate *Arthrobacter* species. Further analysis revealed the presence of up to two copies of the redox-sensitive transcriptional activator gene, *soxR* in isolates H14, H20 and H5. However, the regulatory gene, *soxS* was absent in all Antarctic and temperate *Arthrobacter* genomes. In *E. coli*, SoxR regulates the expression of transcription factor, SoxS in response to H_2_O_2_ and other superoxide compounds. SoxS in turn activates the expression of several superoxide stress response genes. SoxR is observed without SoxS in many organisms including *Pseudomonas aeruginosa*, *P. putida* and *Streptomyces coelicolor*. For these bacteria, it is hypothesized that the SoxR homolog regulates the redox-active secondary metabolite, pyocyanin, which is involved in redox homeostasis [41]. However, as genomes of the Antarctic *Arthrobacter* strains lack homologs of genes involved in phenazine biosynthesis, we hypothesize that the SoxR regulon may directly interact with proteins involved in the superoxide stress response.

#### Osmotic stress response

Metabolites including compatible solutes, cryoprotectants, exopolysaccharides (EPS) and other protective polysaccharides can confer resistance to environmental stressors including UV radiation, osmotic stress, oxidation, and desiccation, thus playing a crucial role in the Antarctic soil environment. Glycogen and trehalose protect the cell from desiccation, osmotic stress and cold shock, and under nutrient limiting conditions can also serve as a source of carbon [42]. The glycogen biosynthesis pathway (from ADP-D-glucose) comprises three steps catalyzed by three enzymes, glucose-1-phosphate adenylytransferase (*glgC*), ADP-glucose type (glycosyl-transferring) starch/glycogen synthase (*glgA*) and glycogen branching enzyme (*glgB*). Genes (*glgA*, *glgB* and *glgC*) for the entire glycogen biosynthesis pathway were identified in all Antarctic and temperate *Arthrobacter* genomes. Genes encoding glycogen degradation enzymes, glucan phosphorylase (*glgP*) and glycogen debranching enzyme (*glgX*), were also identified in all Antarctic and temperate *Arthrobacter* genomes [43]. Genes for trehalose biosynthesis, trehalose-6-phosphate synthase (*otsA*) and trehalose-6-phosphate phosphatase (*otsB*) were present in all Antarctic and temperate *Arthrobacter* genomes. In *E. coli*, trehalose synthesis by enzymes OtsA and OtsB is induced by cold shock and is essential for cell viability [44]. In the psychrotolerant bacterium *Arthrobacter* strain A3, trehalose serves as a source of carbon, allowing cells to maintain normal metabolism at prolonged low temperatures [45]. Additional pathways for trehalose biosynthesis from maltose by the enzyme trehalose synthase (*treS*), and from maltodextrin by malto-oligosyl trehalose synthase (*treY*) and malto-oligosyl trehalose trehalohydrolase (*treZ*), were also identified in all Antarctic and temperate *Arthrobacter* genomes. Genome analysis has also revealed the presence of genes encoding a number of ABC-type transporter systems for mediating cytoplasmic accumulation of organic compatible solutes including choline, glycine betaine and proline (*proV*, *proW, proX*) [46] in all Antarctic and temperate *Arthrobacter* genomes. Genome surveys of psychrophilic bacteria and archaea also revealed the presence of multiple genes for the uptake and/or synthesis of compatible solutes, illustrating the importance of these compounds for osmoprotection and cryoprotection in cold environments [47,48].

A large number of genes involved in several sugar biosynthesis pathways were identified in all Antarctic and temperate *Arthrobacter* genomes (Additional file 7). EPS produced by members of the genera *Pseudoalteromonas*, *Shewanella*, *Polaribacter*, and *Flavobacterium* isolated from the Antarctic marine environment was largely composed of neutral sugars including glucose, fucose, and mannose, amino sugars including N-acetyl galactosamine and N-acetyl glucosamine and uronic acids including galacturonic and glucuronic acids. In these bacteria, EPS may protect cells from the extremes of low temperature and high salinity in the marine environment [49]. EPS production has also been reported in psychrophilic bacteria such as *C. psychrerythraea* [39] and *Psychromonas ingrahamii* [36]. The presence of a wide range of osmoprotection systems suggests that the Antarctic *Arthrobacter* isolates are well equipped to cope with desiccation and osmotic stress.

#### Cold shock response

Cold shock can result in the inhibition of bacterial cell growth and proliferation as a result of stabilization of DNA and RNA secondary structures, reduction in membrane fluidity and solute uptake. Therefore, upon exposure to a sudden temperature downshift, bacteria respond with a specific pattern of transient gene expression of members of a family of small, nucleic acid-binding cold-shock proteins (CSPs). CSPs are of note as they regulate messenger RNA (mRNA) translation, rate of mRNA degradation and transcription termination, all of which are dependent on the ribosome that is targeted by cold shock [50-52]. Several copies of the DNA-binding cold-shock proteins were identified in all Antarctic and temperate *Arthrobacter* genomes similar to the multiple copies observed in C. *psychrerythraea* [39], *Psychrobacter arcticus* [53] and *Shewanella oneidensis* [54]. Additionally, genes encoding cold-shock-inducible proteins: ribosome-binding factor A (*rbfA*), translation initiation factors, IF-1 and IF-2 (*infA*, *infB*), polynucleotide phosphorylase (*pnp*), RNA-binding cold-shock domain A (*csdA*) and NusA, N-using substance protein A (*nusA*) were identified in the Antarctic and temperate *Arthrobacter* genomes [55]. Homologs of the CspA-like cold acclimation protein, CapA, were identified in genomes of four Antarctic *Arthrobacter* isolates, 35/47, H14, H5 and I3. Unlike CSPs that are transiently expressed upon cold shock, in *A. globiformis* S155, CapA was over-expressed following cold shock and during prolonged growth at low temperatures [51]. The presence of the CapA homolog in Antarctic *Arthrobacter* genomes may explain the survival of these organisms at prolonged low temperatures. Genes for CapA were also identified in genomes of all temperate *Arthrobacter* species.

#### Cell membrane adaptations

In the Antarctic soil environment, maintaining membrane permeability and fluidity at sub-zero temperatures is crucial for continued cell viability. Genome analyses of Antarctic *Arthrobacter* isolates have revealed the presence of several genes for fatty acid desaturases (*des*), which are important in this context. Upon cold shock, the expression of *des* genes is regulated by a two-component signal transduction pathway, comprising a membrane-integrated histidine kinase that senses change in membrane fluidity and a response regulator that binds the promoter region of *des* genes. Together they increase the production of desaturases that add double bonds into pre-existing fatty acid tails within the membrane, thus restoring membrane fluidity. Genes for desaturases were also observed in genomes of all temperate *Arthrobacter* spp., similar to the copies observed in the psychrotolerant bacterium, *Exiguobacterium sibiricum* [56]. In *Bacillus subtilis*, the DesKR system regulates the expression of the *des* gene coding for ∆5 desaturase [57,58]. All Antarctic *Arthrobacter* isolates contained several copies of genes associated with signal transduction mechanisms (approx. 3% of the total CDSs) (Additional file 8). A similar proportion of genes (approx. 4% of the total CDSs) were attributed to signal transduction mechanisms in genomes of the temperate *Arthrobacter* species.

Carotenoid biosynthesis also contributes to cold adaptation by stabilizing the cell membrane, maintaining proton permeability and by promoting oxidative stress resistance [59,60]. Pigmentation is a common feature of Antarctic *Arthrobacter* isolates. Genes involved in the synthesis of lycopene (red-coloured) have been identified in Antarctic *Arthrobacter* strains H41 and Br18. Decaprenoxanthin (yellow-coloured) biosynthesis genes have been identified in Antarctic *Arthrobacter* strains H14, H20, and 35/47 [61]. These findings are in agreement with the coloured pigments produced by these isolates (data not shown). Genome analysis of *P. halocryophilus* also revealed the presence of genes for lycopene biosynthesis. It is hypothesized that these genes are responsible for the bright orange colouration observed in *P. halocryophilus* cultures [35]. The decaprenoxanthin biosynthesis pathway includes genes, *crtE* (geranylgeranyl pyrophosphate synthase), *crtI* (phytoene desaturase) and *crtB* (phytoene synthase) for the production of lycopene and genes, *crtEB* (lycopene elongase) and *crtYef* (lycopene epsilon cyclise) for the production of decaprenoxanthin. Antarctic *Arthrobacter* isolates I3 and H5 only contained copies of genes *crtE* and *crtI*. In contrast, just two of the seven temperate *Arthrobacter* spp., *A. aurescens* TC1 and *A. castelli* DSM 16402, contained genes involved in decaprenoxanthin biosynthesis.

### Phenotypic characterisation of Antarctic *Arthrobacter* strains

Seven Antarctic *Arthrobacter* isolates and three temperate, soil-dwelling *Arthrobacter* species were selected for phenotypic characterisation and comparative analysis by BIOLOG PM1 and PM2A plates assessing carbon utilisation and plate PM9 assessing salinity tolerance.

#### Carbon utilisation profiles

**Figure 6:**
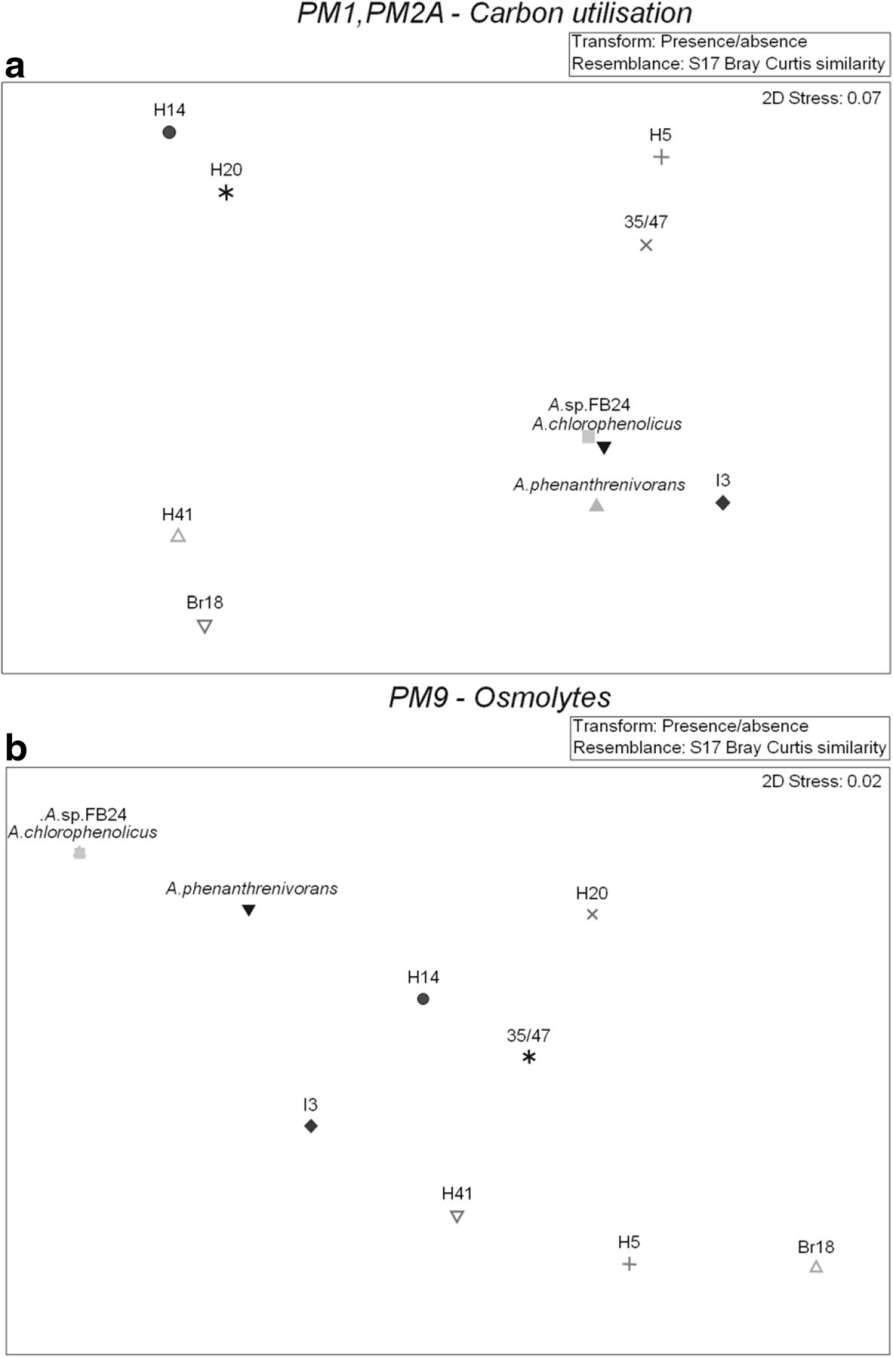
**MDS plots of BIOLOG substrate usage data of seven Antarctic** ***Arthrobacter*** **isolates and three closely related temperate** ***Arthrobacter*** **species. a**. Carbon utilisation profile determined by BIOLOG PM1 and PM2A assay plates. **b**. Salinity tolerance profile determined by BIOLOG PM9 assay plate. Antarctic strains: I3, H14, H5, H20, 35/47, Br18, H41. Temperate strains: *A. chlorophenolicus* A6, *A. phenathrenivorans* Sphe3, *Arthrobacter* sp. FB24.

Antarctic *Arthrobacter* isolates demonstrated significantly lower metabolic versatility as compared to temperate *Arthrobacter* isolates (P <0.05), as the temperate species were able to utilise 123-140 different C sources and the Antarctic strains were only able to utilise 98-121 different C sources (Additional file 9). The diverse patterns of C utilisation were further reflected in the MDS plot as four separate clusters were observed (Figure 6a).

Antarctic *Arthrobacter* isolates were able to utilise C substrates that form components of bacterial and fungal cell walls [62,63] including carbohydrates, N-acetyl glucosamine, glucose, mannose, xylose, arabinose, rhamnose and amino acids, Ala, Glu, Gly, and Lys, glycogen and trehalose that serve as microbial carbon and energy storage molecules [43,64] and sucrose, mannitol and arabitol that serve as storage molecules in lichens [65]. With the exception of ornithogenic soils formed under penguin rookeries, soils of the RSR typically contain low concentrations of organic C, ranging from 0.01 to 0.96 mg C g^−1^ soil [66]. Recalcitrant compounds including pectin and lignin are limited in Antarctic soil environments due to the absence of higher plants [67]. Instead, Antarctic soil environments largely contain C compounds as a result of aeolian distribution of organic C derived predominantly from: (i) cyanobacterial mats and mosses from lacustrine and marine ecosystems, (ii) endolithic microbial communities, and (iii) soil-dwelling mosses, lichens and microbial communities [66]. In addition, the Antarctic isolates lacked the ability to utilise plant cell wall components including carbohydrate, allose; carboxylic acids, citric acid, 4-hydroxybenzoic acid and galacturonic acid; and polymer, pectin, all of which are typically absent in RSR soils [68]. In contrast, all three temperate *Arthrobacter* spp. were able to utilise these C sources.

#### Salinity tolerance patterns

Antarctic *Arthrobacter* isolates showed a significantly narrower salinity tolerance range as compared to temperate *Arthrobacter* species (P <0.01) (Additional file 10). Differences in salinity tolerance are clearly illustrated in the MDS plot where two separate clusters of temperate and Antarctic *Arthrobacter* spp. are observed (Figure 6b). A positive phenotype for all Antarctic *Arthrobacter* isolates was observed with up to 7% NaCl, 4% KCl and 5% sodium sulphate and up to 100 mM ammonium sulphate (pH 8) and 100 mM sodium nitrate. Salinity is a key feature of soils in the RSR with some soils, such as those at lower Taylor Valley, containing 62.04 kg of salts m^-2^ [69]. The salt composition of RSR soils is largely dependent on geographic location as coastal soils are largely comprised of chloride and nitrate salts while inland soils are dominated by sulphate and nitrate salts [67]. As a result, an ability to withstand high concentrations of these salts is essential for survival in the soil environment. As compared to temperate *Arthrobacter* spp., all Antarctic strains showed significantly reduced tolerance to organic salts, Na formate (<1%) and Na lactate (<1%), which are likely to be absent in Antarctic soil environments.

## Conclusions

We have undertaken a comparative genomic study of seven Antarctic and seven temperate *Arthrobacter* spp. to identify genomic features that may be essential for growth and survival in the Antarctic terrestrial environment. Genomes of all Antarctic *Arthrobacter* isolates contained several features that are also observed in psychrophilic/psychrotolerant bacteria and archaea. These included genes for sigma factors, ROS detoxification, osmoprotection systems, cold shock response and a carotenoid biosynthesis pathway. However, a large proportion of these genes were also identified in temperate *Arthrobacter* spp., suggesting that these genes may be important for growth and survival in a range of soil habitats. Further investigations by transcriptomic- and proteomic-based techniques, as previously reported for *A. chlorophenolicus* [70,71] and *A. phenanthrenivorans* [72], are essential to reveal the expression profiles of these genes as well as identify novel traits or genes that are crucial for cold adaptation.

It should be noted that relative to temperate species, and notwithstanding the incompleteness of the genome assemblies, four Antarctic *Arthrobacter* isolates (Br18, H20, H41 and 35/47) contained significantly fewer CDSs. Phenotypic characterisation assessing carbon utilisation profiles of these isolates revealed lowered metabolic versatility. In addition, of the total CDSs identified in the genomes of these isolates significantly fewer CDSs were assigned to COG categories, transcription [K] and carbohydrate transport and metabolism [G] (P < 0.01). The fewer CDSs, the lowered metabolic versatility, and the significant reduction in CDSs associated with transcription and carbohydrate transport and metabolism, suggest the occurrence of genome content scaling in four of the seven Antarctic *Arthrobacter* isolates. This occurrence was also reported in three strains of *Paenibacillus darwinianus* that were isolated from gamma-irradiated soils of the RSR [73,74]. In bacteria, an increase in genome size is often linked with an increase in metabolic versatility, allowing bacteria to produce new enzymes that exploit a wide range of environmental conditions [75]. However, an increase in versatility is linked with a four-fold increase in regulatory proteins associated with transcription and two-component signal transduction systems [76,77]. In environments such as soil, efficient regulation of enzyme expression, enabling exploitation of scarce yet diverse, complex nutrients can offer a selective advantage, thus lowering the penalty of slow growth. This growth strategy is common amongst dominant bacteria in soil environments [78]. In the Antarctic soil environment, organic residues are scarce yet labile, with C and N being mineralisable within a relatively short period of time (90 days) under optimal conditions [79]. In the harsh Antarctic soil environment, maintenance of metabolic versatility comes at a higher cost and, more importantly, reproductive efficiency (promoted by smaller genomes containing fewer CDSs) is beneficial for survival and growth. Finally, these genome sequences allow further investigations into the expression of physiological traits that enable survival under extreme conditions and, more importantly, into the ability of these bacteria to respond to future perturbations including climate change and human impacts.

## Materials and methods

### qPCR analysis

#### Site descriptions and sampling strategy

Soil samples were collected from four sites: Scott Base (77°55’S, 166°45’E), Granite Harbour (77°24’S, 162°31’E), Minna Bluff (78°31’S, 166°46’E) and Marble Point (77°25’S, 163°41’E). To the best of our knowledge all sites sampled were free of recent anthropogenic disturbances, with a possible exception of foot traffic. Soil classification and descriptions are as previously described [80]. At each site, three pits (_~_ 50 m from each other) representing biological replicates were dug. Following removal of the desert pavement, four soil samples (_~_ 15 g each), two samples at depths 0-5 cm and two at 5-10 cm, representing technical replicates were collected. Soil samples were stored in LifeGuard™ Soil Preservation Solution (MoBio), 2 ml of solution per g soil, frozen at -20°C and transported back to New Zealand for processing. For long-term storage, samples were frozen at -80°C.

#### DNA extraction

DNA was extracted in duplicate (0.5 g soil/tube) from one technical replicate at each depth at all sample sites by mechanical cell disruption (bead-beating) as previously described [81]. DNA extracts were quantified by Qubit® dsDNA BR assay kit (Life Technologies) and their purity (A_260_/A_280_) was assessed on a NanoDrop™ ND-1000 Spectrophotometer (Biolab).

#### qPCR validation and protocol

qPCR assays were designed for the selective amplification of: (1) all *Bacteria*, (2) phylum *Actinobacteria*, (3) genus *Arthrobacter*. Primer sequences for each reaction are listed in Additional file 11. Specificity of all primer sequences was tested *in silico* using the Probe Match tool in the RDP-Release 10 (Ribosomal Database Project) classifier program [82]. Primer reactions were optimised with genomic DNA from the following pure cultures: *Arthrobacter* spp. H5, H14, H20 and 35/47, *Nocardioides* sp. D26, *Pontibacter* sp. D8, *Modestobacter* sp. Br44, *Paenibacillus darwinianus* Br^T^, and *Escherichia coli* NZRM 916. Finally, primer specificity was tested by clone library preparations as described by Aislabie *et al*. [8] with DNA from: three Antarctic soil samples (Scott Base, Minna Bluff and Marble Point) and a marine sponge sample (*Rhopaloeides odorabile*). Twenty clones per DNA sample were selected for identification by 16S rRNA gene sequencing.

All qPCR reactions were performed in 10 μl reaction volumes in 384-well clear optical reaction plates (Applied Biosystems) on a 7900HT Fast Real-Time PCR System (Applied Biosystems). Each reaction contained: 5 μl of Platinum® Green qPCR SuperMix-UDG with ROX (Life Technologies), 0.2 μM forward and reverse primers for all *Bacteria* and *Actinobacteria*-specific assays; 0.5 μM forward and reverse primers for *Arthrobacter*-specific assays*,* 1 μl template DNA (1 ng/μl) and ddH_2_O up to 10 μl. General qPCR cycling conditions were 2 min at 50°C, 2 min at 95°C, followed by 40 cycles of 95°C for 15 s and 1 min at the respective annealing temperature. Annealing temperatures were individually optimized for each assay. Each plate included triplicate reactions for all DNA samples, DNA standards and no-template controls. Melting curve analysis was performed at the end of each reaction to confirm the attribution of the fluorescence signal to a specific PCR product and not primer-dimers or other artifacts. DNA standards were prepared from a linear plasmid containing the entire 16S rRNA gene, amplified and cloned as described by Aislabie *et al*. [8] from genomic DNA of *Arthrobacter* sp. FB24 (NR_074590).

Standard curves and amplification efficiencies were calculated using 7900HT SDS software version 2.4 as described elsewhere [83]. Correlation coefficient (R^2^) and amplification efficiency (E) across all qPCR assays were >0.99 and 1.80-1.86, respectively.

### Phylogenetic analyses of *Arthrobacter* from soils of the RSR

#### Phylogenetic analysis

Nearly full-length 16S rRNA gene sequences of *Arthrobacter* spp. from soils of the RSR were obtained from GenBank, aligned via the SINA web aligner and imported into the ARB phylogenetic package using the SILVA 108 database for analysis by the maximum likelihood, RAxML method. The topology of the tree was tested in MEGA v6.0 by bootstrap analysis based on 1000 resamplings [84,85].

#### Selection of isolates

Seven *Arthrobacter* isolates from RSR soils including: I3, H5, H14, H20, 35/47, Br18 and H41 were obtained from the Antarctic culture collection of Dr Jackie Aislabie (Landcare Research, Hamilton). These isolates were routinely cultured on R2A agar (Difco™, BD) at 15°C. Temperature range data for isolate 35/47 were determined by growth on R2A plates for two weeks at a range of temperatures, namely 5, 10, 15, 18, 20, 25, and 37°C.

#### DNA extraction and sequencing

High molecular weight DNA was extracted from seven *Arthrobacter* isolates: I3, H5, H14, H20, 35/47, Br18 and H41 using a modified CTAB (hexadecyltrimethylammonium bromide) and protein lysis method [86]. Briefly, cells were scraped off R2A agar plates and re-suspended in 740 μl TE buffer containing 20 μl lysozyme (100 mg/ml), 40 μl 10% SDS and 8 μl proteinase K (20 mg/ml). These cells were incubated overnight at 37°C. Following overnight incubation, 100 μl of 5 M NaCl and CTAB/NaCl solutions were added to the reaction and incubated at 65°C for 10 min. Subsequently 0.5 ml chloroform:isoamyl alcohol (24:1) was added, and the entire reaction was centrifuged at 16,000 g for 15 min. The aqueous phase was transferred to a clean microcentrifuge tube containing 0.5 ml phenol:chloroform:isoamyl alcohol (25:24:1) and the reaction was centrifuged at 16,000 g for 15 min. The aqueous phase was transferred to a clean microcentrifuge tube containing 0.6 vol isopropanol. To allow for DNA precipitation, all reactions were incubated at room temperature for 60 min, then centrifuged at 16,000 g for 30 min. The resulting DNA pellet was washed with 70% ethanol and re-suspended in TE buffer containing RNAse (99 μl TE buffer + 1 μl RNAse (10 mg/ml)) and incubated at 37°C for 20 min. DNA extracts were quantified by Quant-iT™ PicoGreen® dsDNA assay kit (Life Technologies) and their purity (A_260_/A_280_) was assessed on a NanoDrop™ ND-1000 Spectrophotometer (Biolab). Additionally, quality of each DNA extract was tested by electrophoresis on a 1% agarose gel.

Following extraction, high molecular weight DNA was sent to Macrogen (Seoul, South Korea) for sequencing on the Illumina HiSeq 2000 platform using 100 bp paired end libraries. With a sequencing output of 35 Gb, estimated coverage was 700X per genome.

#### *De novo* assembly, annotation and comparative analyses

FASTQ files for each genome were trimmed and quality filtered using the FASTQ Trimmer tool of the FASTX-toolkit v0.0.13 [87] and Sickle (https://github.com/ucdavis-bioinformatics/sickle) respectively. High-quality reads (Q > 30) were assembled into contigs using Velvet v1.2.10 [88]. Following initial assembly, PAGIT (post assembly genome improvement toolkit) tools including IMAGE (iterative mapping and assembly for gap elimination) and iCORN (iterative correction of reference nucleotides) were utilized for gap elimination and sequencing error correction [89]. Finally, SSPACE basic version 1.0 (stand-alone scaffolder of pre-assembled contigs using paired-read data) was utilized to build scaffolds from assembled contigs [90]. Genome completeness was assessed as previously described [91]. Gene prediction and genome annotation was performed using the automated JGI pipeline (Joint Genome Institute) [92].

For comparative analyses, *A. aurescens* TC1, *A. castelli* DSM 16402, *A. chlorophenolicus* A6, *A. globiformis* NBRC 12137, *A. nitroguajacolicus* Rue61a, *A. phenanthrenivorans* Sphe3 and *Arthrobacter* sp. FB24 were selected based on their genome completeness and habitat. These analyses were performed using JGI-IMG (Integrated Microbial Genomes)-Expert Review [85]. CMG biotools was utilised for pan- and core-genome plot analysis and predicted proteome comparisons [32]. Putative phage sequences were identified by the Phage Search Tool (PHAST) [24].

#### Availability of supporting data

This whole genome shotgun project has been deposited at DDBJ/EMBL/GenBank under the following accession numbers: 35/47-AZHY00000000, H14-AZRX00000000, H20-AZRY00000000, H41-AZRZ00000000, Br18-AZSA00000000, I3-AZSB00000000 and H5-AZSC00000000.

### Phenotypic characterisation by BIOLOG

#### Salinity tolerance and carbon substrate utilisation

Carbon utilisation (PM1, PM2A) and salinity tolerance (PM9) were tested by Phenotype Microarray (PM) technology (BIOLOG). Ten *Arthrobacter* isolates, including seven Antarctic isolates: I3, H5, H14, H20, 35/47, Br18 and H41 and three temperate isolates: *A. phenanthrenivorans* DSM 18606 ^T^, *A. chlorophenolicus* DSM 12829 ^T^ and *Arthrobacter* sp. FB24 DSM 22572^T^, were included for characterisation. Type strains of the temperate bacteria were obtained from DSMZ, Germany. Antarctic isolates were routinely cultured on R2A agar plates at 15°C and temperate isolates were routinely cultured on TSB (tryptic soy broth) agar (Bacto™, BD) plates at 28°C. Prior to inoculation of PM plates, Antarctic isolates were grown at 15°C for three days on R2A agar plates and temperate isolates were grown at 28°C for 12 h on TSB agar plates. PM plates were inoculated with 150 μl of cell suspension, prepared to the appropriate cell density as per the manufacturer’s instructions. PM plates inoculated with Antarctic isolates were incubated at 15°C and PM plates with temperate isolates were incubated at 28°C. Colour development (OD_600nm_) on all plates was recorded at 24 h intervals on an EnSpire® multimode plate reader over 10 days for Antarctic isolates and over four days for temperate isolates.

#### Data analysis

Isolate response profiles were built based on the response of each isolate to each substrate, determined as either positive or negative (binary data – usage/non-usage). Response profiles were analysed with the multivariate statistics package, Primer-E v6 (UK) by non-metric multi-dimensional scaling (MDS) plots on Bray Curtis similarity matrices constructed from transformed data (presence/absence).

## Additional files

Additional file 1:
**Phylogenetic tree based on nearly complete 16S rRNA gene sequences of** ***Arthrobacter*** **isolates and clones from RSR soils, constructed by the maximum likelihood, RAxML method.**
Additional file 2:
**List of mobile genetic element associated genes in Antarctic and temperate** ***Arthrobacter*** **genomes.**
Additional file 3:
**Putative phage sequences identified in Antarctic** ***Arthrobacter*** **strains Br18 (a, b); H20 (c); H14 (d) by phage search tool, PHAST [24].**
Additional file 4:
**The core- and pan- genome of 14** ***Arthrobacter*** **genomes calculated using BLAST in CMG Biotools.**
Additional file 5:
**List of genes linked to environmental stress response in Antarctic and temperate** ***Arthrobacter*** **genomes.**
Additional file 6:
**Putative oxidases identified in seven temperate and seven Antartic** ***Arthrobacter*** **genomes.**
Additional file 7:
**Number of genes associated with sugar biosynthesis pathways in temperate and Antarctic** ***Arthrobacter*** **genomes.**
Additional file 8:
**Genes associated with histidine kinase signal transduction systems identified in temperate and Antarctic** ***Arthrobacter*** **genomes.**
Additional file 9:
**Carbon utilisation profiles of three temperate** ***Arthrobacter*** **spp. and seven Antarctic** ***Arthrobacter*** **isolates determined by BIOLOG PM1 and PM2A carbon plates.**
Additional file 10:
**Salinity tolerance profiles of three temperate** ***Arthrobacter*** **spp. and seven Antarctic** ***Arthrobacter*** **isolates determined by the BIOLOG PM9 Osmolyte plate.**
Additional file 11:
**A description of taxon-specific primers used for qPCR assays.**

## Abbreviations

RSR: Ross sea region
qPCR: quantitative polymerase chain reaction
OTU: Operational taxonomic unit
CDS: Protein coding sequence
COG: Clusters of orthologous groups
TCS: Two-component systems
ROS: Reactive oxygen species
ABC: ATP-binding cassette
