# Supplementary material for: Genomic and phenotypic insights into the ecology of Arthrobacter from Antarctic soils
Source: BMC Genomics. 2015 Feb 5;16(1):36. doi: 10.1186/s12864-015-1220-2 (PMC4326396; doi:10.1186/s12864-015-1220-2)
Supplement: Additional file 1: — Phylogenetic tree based on nearly complete 16S rRNA gene sequences of Arthrobacter isolates and clones from RSR soils, constructed by the maximum likelihood, RAxML method. [file 12864_2015_1220_MOESM1_ESM.pptx]

## Slide 1
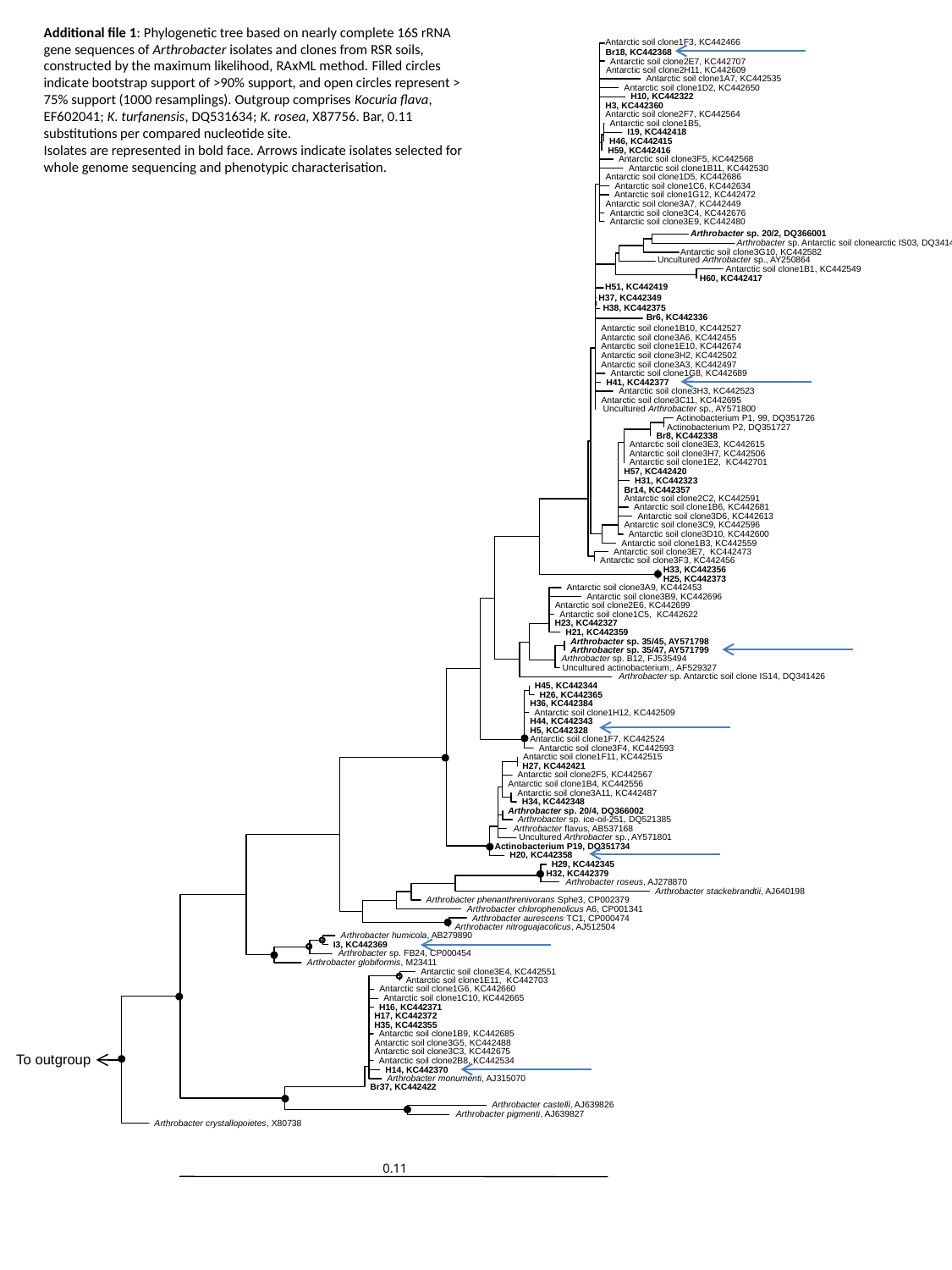

Additional file 1: Phylogenetic tree based on nearly complete 16S rRNA gene sequences of Arthrobacter isolates and clones from RSR soils, constructed by the maximum likelihood, RAxML method. Filled circles indicate bootstrap support of >90% support, and open circles represent > 75% support (1000 resamplings). Outgroup comprises Kocuria flava, EF602041; K. turfanensis, DQ531634; K. rosea, X87756. Bar, 0.11 substitutions per compared nucleotide site.
Isolates are represented in bold face. Arrows indicate isolates selected for whole genome sequencing and phenotypic characterisation.
Antarctic soil clone1F3, KC442466
Br18, KC442368
Antarctic soil clone2E7, KC442707
Antarctic soil clone2H11, KC442609
Antarctic soil clone1A7, KC442535
Antarctic soil clone1D2, KC442650
H10, KC442322
H3, KC442360
Antarctic soil clone2F7, KC442564
Antarctic soil clone1B5,
I19, KC442418
H46, KC442415
H59, KC442416
Antarctic soil clone3F5, KC442568
Antarctic soil clone1B11, KC442530
Antarctic soil clone1D5, KC442686
Antarctic soil clone1C6, KC442634
Antarctic soil clone1G12, KC442472
Antarctic soil clone3A7, KC442449
Antarctic soil clone3C4, KC442676
Antarctic soil clone3E9, KC442480
Arthrobacter sp. 20/2, DQ366001
Arthrobacter sp. Antarctic soil clonearctic IS03, DQ341415
Antarctic soil clone3G10, KC442582
Uncultured Arthrobacter sp., AY250864
Antarctic soil clone1B1, KC442549
H60, KC442417
H51, KC442419
H37, KC442349
H38, KC442375
Br6, KC442336
Antarctic soil clone1B10, KC442527
Antarctic soil clone3A6, KC442455
Antarctic soil clone1E10, KC442674
Antarctic soil clone3H2, KC442502
Antarctic soil clone3A3, KC442497
Antarctic soil clone1G8, KC442689
H41, KC442377
Antarctic soil clone3H3, KC442523
Antarctic soil clone3C11, KC442695
Uncultured Arthrobacter sp., AY571800
Actinobacterium P1, 99, DQ351726
Actinobacterium P2, DQ351727
Br8, KC442338
Antarctic soil clone3E3, KC442615
Antarctic soil clone3H7, KC442506
Antarctic soil clone1E2,  KC442701
H57, KC442420
H31, KC442323
Br14, KC442357
Antarctic soil clone2C2, KC442591
Antarctic soil clone1B6, KC442681
Antarctic soil clone3D6, KC442613
Antarctic soil clone3C9, KC442596
Antarctic soil clone3D10, KC442600
Antarctic soil clone1B3, KC442559
Antarctic soil clone3E7,  KC442473
Antarctic soil clone3F3, KC442456
H33, KC442356
H25, KC442373
Antarctic soil clone3A9, KC442453
Antarctic soil clone3B9, KC442696
Antarctic soil clone2E6, KC442699
Antarctic soil clone1C5,  KC442622
H23, KC442327
H21, KC442359
Arthrobacter sp. 35/45, AY571798
Arthrobacter sp. 35/47, AY571799
Arthrobacter sp. B12, FJ535494
Uncultured actinobacterium,, AF529327
Arthrobacter sp. Antarctic soil clone IS14, DQ341426
H45, KC442344
H26, KC442365
H36, KC442384
Antarctic soil clone1H12, KC442509
H44, KC442343
H5, KC442328
Antarctic soil clone1F7, KC442524
Antarctic soil clone3F4, KC442593
Antarctic soil clone1F11, KC442515
H27, KC442421
Antarctic soil clone2F5, KC442567
Antarctic soil clone1B4, KC442556
Antarctic soil clone3A11, KC442487
H34, KC442348
Arthrobacter sp. 20/4, DQ366002
Arthrobacter sp. ice-oil-251, DQ521385
Arthrobacter flavus, AB537168
Uncultured Arthrobacter sp., AY571801
Actinobacterium P19, DQ351734
H20, KC442358
H29, KC442345
H32, KC442379
Arthrobacter roseus, AJ278870
Arthrobacter stackebrandtii, AJ640198
Arthrobacter phenanthrenivorans Sphe3, CP002379
Arthrobacter chlorophenolicus A6, CP001341
Arthrobacter aurescens TC1, CP000474
Arthrobacter nitroguajacolicus, AJ512504
Arthrobacter humicola, AB279890
I3, KC442369
Arthrobacter sp. FB24, CP000454
Arthrobacter globiformis, M23411
Antarctic soil clone3E4, KC442551
Antarctic soil clone1E11,  KC442703
Antarctic soil clone1G6, KC442660
Antarctic soil clone1C10, KC442665
H16, KC442371
H17, KC442372
H35, KC442355
Antarctic soil clone1B9, KC442685
Antarctic soil clone3G5, KC442488
To outgroup
Antarctic soil clone3C3, KC442675
Antarctic soil clone2B8, KC442534
H14, KC442370
Arthrobacter monumenti, AJ315070
Br37, KC442422
Arthrobacter castelli, AJ639826
Arthrobacter pigmenti, AJ639827
Arthrobacter crystallopoietes, X80738
0.11
