# Supplementary material for: Genomic and phenotypic insights into the ecology of Arthrobacter from Antarctic soils
Source: BMC Genomics. 2015 Feb 5;16(1):36. doi: 10.1186/s12864-015-1220-2 (PMC4326396; doi:10.1186/s12864-015-1220-2)
Supplement: Additional file 3: — Putative phage sequences identified in Antarctic Arthrobacter strains Br18 (a, b); H20 (c); H14 (d) by phage search tool, PHAST [24]. [file 12864_2015_1220_MOESM3_ESM.pptx]

## Slide 1
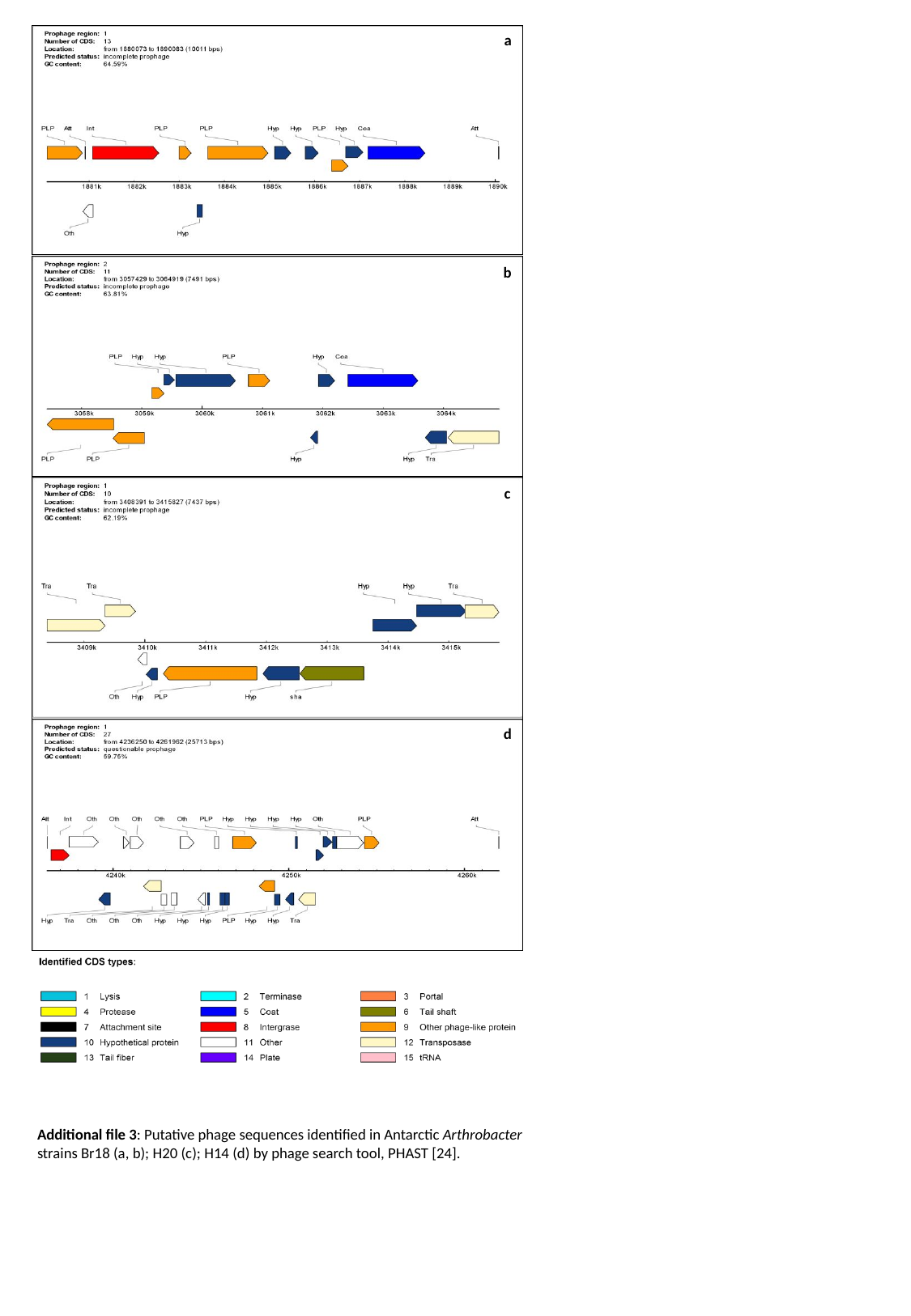

a
b
c
d
Additional file 3: Putative phage sequences identified in Antarctic Arthrobacter strains Br18 (a, b); H20 (c); H14 (d) by phage search tool, PHAST [24].
