# Supplementary material for: Genomic and phenotypic insights into the ecology of Arthrobacter from Antarctic soils
Source: BMC Genomics. 2015 Feb 5;16(1):36. doi: 10.1186/s12864-015-1220-2 (PMC4326396; doi:10.1186/s12864-015-1220-2)
Supplement: Additional file 4: — The core- and pan- genome of 14 Arthrobacter genomes calculated using BLAST in CMG Biotools. [file 12864_2015_1220_MOESM4_ESM.pptx]

## Slide 1
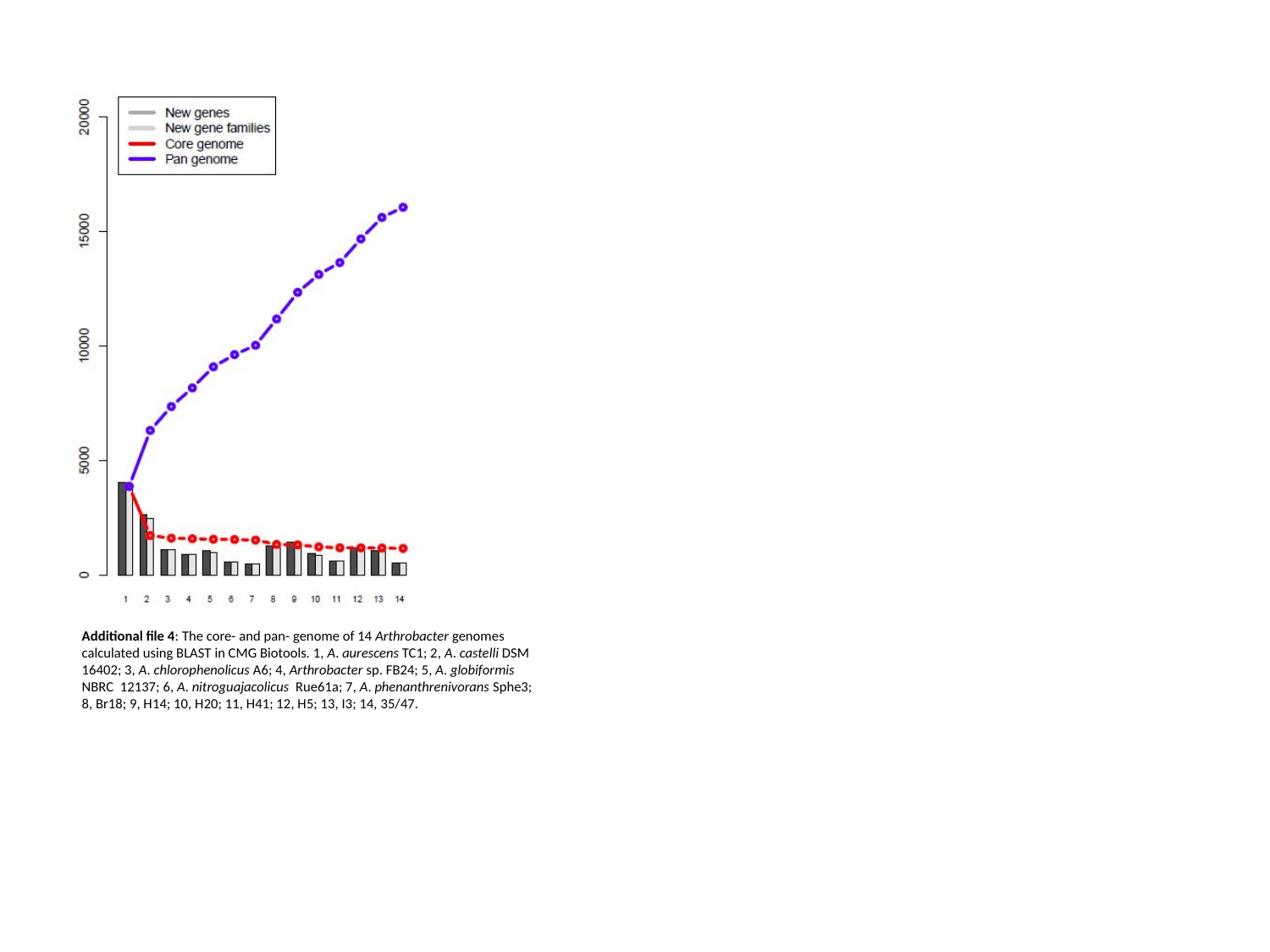

Additional file 4: The core- and pan- genome of 14 Arthrobacter genomes calculated using BLAST in CMG Biotools. 1, A. aurescens TC1; 2, A. castelli DSM 16402; 3, A. chlorophenolicus A6; 4, Arthrobacter sp. FB24; 5, A. globiformis NBRC 12137; 6, A. nitroguajacolicus Rue61a; 7, A. phenanthrenivorans Sphe3; 8, Br18; 9, H14; 10, H20; 11, H41; 12, H5; 13, I3; 14, 35/47.
