# Supplementary material for: Genomic and phenotypic insights into the ecology of Arthrobacter from Antarctic soils
Source: BMC Genomics. 2015 Feb 5;16(1):36. doi: 10.1186/s12864-015-1220-2 (PMC4326396; doi:10.1186/s12864-015-1220-2)
Supplement: Additional file 9: — Carbon utilisation profiles of three temperate Arthrobacter spp. and seven Antarctic Arthrobacter isolates determined by BIOLOG PM1 and PM2A carbon plates. [file 12864_2015_1220_MOESM9_ESM.docx]

Additional file 9: Carbon utilisation profiles of three temperate *Arthrobacter* spp. and seven Antarctic *Arthrobacter* isolates determined by BIOLOG PM1 and PM2A carbon plates.

|  | *A.phenanthrenivornas* | *A.chlorophenolicus* | *A*. sp. FB24 | I3 | H14 | H5 | 35/47 | H20 | H41 | Br18 |
| --- | --- | --- | --- | --- | --- | --- | --- | --- | --- | --- |
| **Amines/Amides** |  |  |  |  |  |  |  |  |  |  |
| Putrescine | 1 | 1 | 1 | 1 | 1 | 1 | 1 | 1 | 0 | 0 |
| D-Glucosamine | 1 | 1 | 1 | 1 | 0 | 1 | 0 | 1 | 1 | 1 |
| Succinamic acid | 0 | 0 | 0 | 1 | 0 | 1 | 1 | 1 | 1 | 1 |
| L-Alaninamide | 0 | 0 | 0 | 0 | 1 | 1 | 1 | 1 | 1 | 1 |
| D-Glucosaminic Acid | 0 | 0 | 0 | 0 | 1 | 1 | 0 | 1 | 1 | 1 |
| Glucuronamide | 1 | 1 | 1 | 0 | 0 | 0 | 0 | 0 | 1 | 1 |
| Phenylethylamine | 1 | 1 | 1 | 1 | 0 | 0 | 0 | 0 | 0 | 1 |
| Sec-Butylamine | 1 | 0 | 1 | 1 | 0 | 0 | 0 | 0 | 1 | 1 |
| Tyramine | 0 | 1 | 1 | 1 | 0 | 0 | 0 | 0 | 0 | 0 |
| D,L-Octopamine | 0 | 1 | 1 | 1 | 0 | 0 | 0 | 0 | 0 | 0 |
| 2-Aminoethanol | 1 | 0 | 0 | 0 | 0 | 0 | 0 | 0 | 0 | 0 |
| N-Acetyl-β -D-Mannosamine | 0 | 0 | 0 | 0 | 0 | 0 | 0 | 0 | 0 | 0 |
| Acetamide | 0 | 0 | 0 | 0 | 0 | 0 | 0 | 0 | 0 | 0 |
| **Amino Acids** |  |  |  |  |  |  |  |  |  |  |
| D-Alanine | 1 | 1 | 1 | 1 | 1 | 1 | 1 | 1 | 1 | 1 |
| L-Glutamic acid | 1 | 1 | 1 | 1 | 1 | 1 | 1 | 1 | 1 | 1 |
| L-Glutamine | 1 | 1 | 1 | 1 | 1 | 1 | 1 | 1 | 1 | 1 |
| L-Proline | 1 | 1 | 1 | 1 | 1 | 1 | 1 | 1 | 1 | 1 |
| L-Serine | 1 | 1 | 1 | 1 | 1 | 1 | 1 | 1 | 1 | 1 |
| γ -Amino Butyric acid | 1 | 1 | 1 | 1 | 1 | 1 | 1 | 1 | 1 | 1 |
| L-Phenylalanine | 1 | 1 | 1 | 1 | 1 | 1 | 1 | 1 | 1 | 1 |
| L-Alanine | 1 | 1 | 1 | 1 | 1 | 1 | 1 | 1 | 1 | 0 |
| L-Aspartic Acid | 1 | 1 | 1 | 1 | 1 | 1 | 1 | 1 | 0 | 1 |
| L-Threonine | 1 | 1 | 1 | 1 | 1 | 0 | 1 | 1 | 1 | 1 |
| Glycine | 1 | 1 | 1 | 1 | 1 | 0 | 1 | 1 | 1 | 1 |
| L-Histidine | 1 | 1 | 1 | 1 | 1 | 1 | 1 | 0 | 1 | 1 |
| Glycyl -L -Glutamic Ac id | 1 | 0 | 1 | 1 | 1 | 1 | 1 | 1 | 1 | 0 |
| Glycyl -L -Proline | 1 | 0 | 1 | 1 | 1 | 1 | 0 | 1 | 1 | 1 |
| L-Alanyl Glycine | 1 | 1 | 1 | 1 | 1 | 1 | 1 | 1 | 0 | 0 |
| L-Asparagine | 1 | 1 | 1 | 1 | 1 | 0 | 0 | 1 | 1 | 1 |
| L-Lysine | 1 | 1 | 1 | 1 | 0 | 0 | 1 | 1 | 1 | 1 |
| L-Pyroglutamic acid | 1 | 1 | 1 | 1 | 1 | 1 | 1 | 1 | 0 | 0 |
| L-Leucine | 0 | 0 | 1 | 1 | 1 | 0 | 1 | 1 | 1 | 1 |
| L-Arginine | 1 | 1 | 1 | 1 | 0 | 0 | 1 | 0 | 1 | 1 |
| L-Isoleucine | 1 | 1 | 1 | 1 | 0 | 1 | 1 | 0 | 0 | 0 |
| L-Ornithine | 0 | 0 | 0 | 0 | 0 | 0 | 0 | 1 | 1 | 1 |
| L-Valine | 1 | 0 | 1 | 1 | 0 | 0 | 0 | 0 | 0 | 0 |
| D,L-Carnithine | 1 | 0 | 0 | 1 | 0 | 0 | 0 | 1 | 0 | 0 |
| L-Homoserine | 1 | 0 | 1 | 1 | 0 | 0 | 0 | 0 | 0 | 0 |
| L-Methionine | 0 | 0 | 1 | 1 | 0 | 0 | 0 | 0 | 0 | 0 |
| D-Serine | 0 | 0 | 0 | 1 | 0 | 0 | 0 | 0 | 0 | 0 |
| Glycyl -L -Aspartic acid | 0 | 0 | 0 | 0 | 0 | 0 | 0 | 0 | 0 | 1 |
| Hydroxy-L-Proline | 0 | 0 | 0 | 1 | 0 | 0 | 0 | 0 | 0 | 0 |
| D-Aspartic Acid | 0 | 0 | 0 | 0 | 0 | 0 | 0 | 0 | 0 | 0 |
| D-Threonine | 0 | 0 | 0 | 0 | 0 | 0 | 0 | 0 | 0 | 0 |
| δ-Amino Valeric acid | 0 | 0 | 0 | 0 | 0 | 0 | 0 | 0 | 0 | 0 |
| **Carbohydrates** |  |  |  |  |  |  |  |  |  |  |
| D-Mannitol | 1 | 1 | 1 | 1 | 1 | 1 | 1 | 1 | 1 | 1 |
| D-Mannose | 1 | 1 | 1 | 1 | 1 | 1 | 1 | 1 | 1 | 1 |
| D-Melibiose | 1 | 1 | 1 | 1 | 1 | 1 | 1 | 1 | 1 | 1 |
| D-Ribose | 1 | 1 | 1 | 1 | 1 | 1 | 1 | 1 | 1 | 1 |
| D-Trehalose | 1 | 1 | 1 | 1 | 1 | 1 | 1 | 1 | 1 | 1 |
| D-Xylose | 1 | 1 | 1 | 1 | 1 | 1 | 1 | 1 | 1 | 1 |
| L-Arabinose | 1 | 1 | 1 | 1 | 1 | 1 | 1 | 1 | 1 | 1 |
| L-Lyxose | 1 | 1 | 1 | 1 | 1 | 1 | 1 | 1 | 1 | 1 |
| Maltose | 1 | 1 | 1 | 1 | 1 | 1 | 1 | 1 | 1 | 1 |
| α-D-Glucose | 1 | 1 | 1 | 1 | 1 | 1 | 1 | 1 | 1 | 1 |
| Palatinose | 1 | 1 | 1 | 1 | 1 | 1 | 1 | 1 | 1 | 1 |
| 2-Deoxy-D-Ribose | 1 | 1 | 1 | 1 | 1 | 1 | 1 | 1 | 1 | 1 |
| Dihydroxy acetone | 1 | 1 | 1 | 1 | 1 | 1 | 1 | 1 | 1 | 1 |
| D-Arabinose | 1 | 1 | 1 | 1 | 1 | 1 | 1 | 1 | 1 | 1 |
| Maltitol | 1 | 1 | 1 | 1 | 1 | 1 | 1 | 1 | 1 | 1 |
| α-Methyl-D-Glucoside | 1 | 1 | 1 | 1 | 1 | 1 | 1 | 1 | 1 | 1 |
| Turanose | 1 | 1 | 1 | 1 | 1 | 1 | 1 | 1 | 1 | 1 |
| D-Fructose | 1 | 1 | 1 | 1 | 1 | 1 | 1 | 1 | 0 | 1 |
| L-Rhamnose | 1 | 1 | 1 | 1 | 1 | 0 | 1 | 1 | 1 | 1 |
| Maltotriose | 1 | 1 | 1 | 1 | 1 | 1 | 1 | 1 | 0 | 1 |
| Sucrose | 1 | 1 | 1 | 1 | 1 | 1 | 1 | 1 | 1 | 0 |
| α-Methyl -D-Galactoside | 1 | 1 | 1 | 1 | 1 | 1 | 1 | 1 | 1 | 0 |
| Gentibiose | 1 | 1 | 1 | 1 | 0 | 1 | 1 | 1 | 1 | 1 |
| Arbutin | 1 | 1 | 1 | 1 | 1 | 1 | 1 | 0 | 1 | 1 |
| Lacitol | 1 | 1 | 1 | 1 | 0 | 1 | 1 | 1 | 1 | 1 |
| Stachyose | 1 | 1 | 1 | 1 | 0 | 1 | 1 | 1 | 1 | 1 |
| D-Cellobiose | 1 | 1 | 1 | 1 | 0 | 1 | 1 | 0 | 1 | 1 |
| M-Inositol | 1 | 1 | 1 | 1 | 1 | 0 | 0 | 1 | 1 | 1 |
| β-Methyl-D-Xyloside | 1 | 1 | 1 | 1 | 0 | 1 | 1 | 0 | 1 | 1 |
| D-Melezitose | 1 | 1 | 1 | 1 | 1 | 1 | 1 | 1 | 0 | 0 |
| Melibionic acid | 1 | 1 | 1 | 1 | 1 | 1 | 0 | 0 | 1 | 1 |
| D-Galactose | 1 | 1 | 1 | 1 | 0 | 1 | 1 | 0 | 0 | 1 |
| Lactulose | 1 | 1 | 1 | 1 | 0 | 1 | 1 | 0 | 0 | 1 |
| Xylitol | 1 | 1 | 0 | 1 | 0 | 1 | 1 | 0 | 1 | 1 |
| β-Methyl-D-Galactoside | 0 | 1 | 1 | 1 | 1 | 1 | 1 | 1 | 0 | 0 |
| α-D-Lactose | 1 | 1 | 1 | 1 | 0 | 1 | 1 | 0 | 0 | 0 |
| D-Raffinose | 1 | 1 | 1 | 1 | 0 | 1 | 1 | 0 | 0 | 0 |
| D-Arabitol | 1 | 1 | 0 | 0 | 1 | 1 | 0 | 0 | 1 | 1 |
| D-Sorbitol | 1 | 1 | 0 | 1 | 0 | 1 | 1 | 0 | 0 | 0 |
| N-Acetyl-D-glucosamine | 1 | 0 | 1 | 1 | 0 | 1 | 1 | 0 | 0 | 0 |
| β-Methyl-D-glucoside | 1 | 1 | 1 | 1 | 0 | 1 | 0 | 0 | 0 | 0 |
| D-Tagatose | 1 | 0 | 0 | 0 | 1 | 0 | 0 | 1 | 1 | 1 |
| L-Arabitol | 1 | 0 | 1 | 0 | 0 | 0 | 1 | 0 | 1 | 1 |
| Methyl Pyruvate | 1 | 1 | 1 | 1 | 0 | 0 | 0 | 0 | 0 | 0 |
| N-Acetyl-D-Galactosamine | 0 | 0 | 0 | 0 | 0 | 1 | 0 | 1 | 1 | 1 |
| L-Fucose | 0 | 0 | 0 | 0 | 1 | 0 | 0 | 1 | 1 | 0 |
| D-Fucose | 0 | 0 | 0 | 0 | 1 | 0 | 0 | 0 | 1 | 1 |
| Adonitol | 0 | 0 | 0 | 0 | 1 | 0 | 0 | 1 | 0 | 0 |
| Mannan | 1 | 0 | 0 | 0 | 1 | 0 | 0 | 0 | 0 | 0 |
| L-Sorbose | 0 | 0 | 0 | 0 | 0 | 0 | 0 | 0 | 1 | 1 |
| a-Methyl-D-Mannoside | 0 | 0 | 0 | 0 | 1 | 0 | 0 | 1 | 0 | 0 |
| *β-D-Allose* | 0 | 1 | 1 | 0 | 0 | 0 | 0 | 0 | 0 | 0 |
| D-Psicose | 0 | 0 | 0 | 0 | 0 | 0 | 0 | 0 | 0 | 1 |
| I-Erythritol | 0 | 0 | 0 | 0 | 1 | 0 | 0 | 0 | 0 | 0 |
| Sedoheptulose | 0 | 0 | 0 | 0 | 0 | 0 | 0 | 1 | 0 | 0 |
| 3-Methyl Glucose | 0 | 0 | 0 | 0 | 0 | 0 | 0 | 1 | 0 | 0 |
| Dulcitol | 0 | 0 | 0 | 0 | 0 | 0 | 0 | 0 | 0 | 0 |
| L-Glucose | 0 | 0 | 0 | 0 | 0 | 0 | 0 | 0 | 0 | 0 |
| **Carboxylic acids** |  |  |  |  |  |  |  |  |  |  |
| Acetic Acid | 1 | 1 | 1 | 1 | 1 | 1 | 1 | 1 | 1 | 1 |
| D,L-Malic Acid | 1 | 1 | 1 | 1 | 1 | 1 | 1 | 1 | 1 | 1 |
| D-Gluconic acid | 1 | 1 | 1 | 1 | 1 | 1 | 1 | 1 | 1 | 1 |
| Fumaric acid | 1 | 1 | 1 | 1 | 1 | 1 | 1 | 1 | 1 | 1 |
| Propionic Acid | 1 | 1 | 1 | 1 | 1 | 1 | 1 | 1 | 1 | 1 |
| Succinic Acid | 1 | 1 | 1 | 1 | 1 | 1 | 1 | 1 | 1 | 1 |
| 5-keto-D-Gluconic acid | 1 | 1 | 1 | 1 | 1 | 1 | 1 | 1 | 1 | 1 |
| L-Lactic Acid | 1 | 1 | 1 | 1 | 1 | 1 | 1 | 1 | 1 | 0 |
| L-Malic Acid | 1 | 1 | 1 | 1 | 1 | 1 | 1 | 1 | 0 | 1 |
| Pyruvic acid | 1 | 1 | 1 | 1 | 1 | 1 | 0 | 1 | 1 | 1 |
| Caproic acid | 1 | 1 | 1 | 1 | 1 | 0 | 1 | 1 | 1 | 1 |
| Oxalomalic acid | 1 | 1 | 1 | 0 | 1 | 1 | 1 | 1 | 1 | 1 |
| Glyoxylic Acid | 1 | 1 | 1 | 0 | 1 | 1 | 0 | 1 | 1 | 1 |
| p-Hydroxy Phenyl Acetic Acid | 1 | 1 | 1 | 1 | 1 | 1 | 1 | 1 | 0 | 0 |
| α-Hydroxy Butyric Acid | 1 | 1 | 1 | 0 | 1 | 0 | 1 | 1 | 1 | 1 |
| α-Keto -Butyric Acid | 1 | 1 | 1 | 1 | 1 | 0 | 1 | 1 | 1 | 0 |
| Butryic acid | 1 | 1 | 1 | 1 | 1 | 0 | 0 | 1 | 1 | 1 |
| β-Hydroxy Butyric acid | 1 | 1 | 1 | 1 | 1 | 1 | 1 | 1 | 0 | 0 |
| β-Methyl-D-Glucuronic acid | 1 | 1 | 1 | 0 | 1 | 1 | 0 | 1 | 1 | 1 |
| D-Malic Acid | 1 | 1 | 1 | 1 | 0 | 1 | 1 | 0 | 0 | 1 |
| Citramalic acid | 0 | 1 | 0 | 0 | 1 | 1 | 1 | 1 | 1 | 1 |
| Malonic acid | 0 | 1 | 1 | 1 | 1 | 0 | 0 | 1 | 1 | 1 |
| Quinic acid | 1 | 1 | 1 | 1 | 1 | 0 | 0 | 0 | 1 | 1 |
| Acetoacetic Acid | 1 | 0 | 1 | 0 | 1 | 0 | 0 | 1 | 1 | 1 |
| D-Glucuronic acid | 1 | 1 | 1 | 0 | 0 | 1 | 1 | 0 | 1 | 0 |
| D-Glucuronic acid | 1 | 1 | 1 | 0 | 0 | 1 | 1 | 0 | 1 | 0 |
| Formic Acid | 1 | 1 | 1 | 1 | 0 | 0 | 1 | 0 | 1 | 0 |
| m-Hydroxy Phenyl Acetic Acid | 1 | 1 | 1 | 0 | 1 | 0 | 1 | 1 | 0 | 0 |
| m-Hydroxy Phenyl Acetic Acid | 1 | 1 | 1 | 0 | 1 | 0 | 1 | 1 | 0 | 0 |
| α-Keto -Glutaric Acid | 1 | 1 | 1 | 0 | 1 | 0 | 0 | 1 | 1 | 0 |
| L-Tartaric acid | 0 | 1 | 0 | 1 | 1 | 0 | 1 | 0 | 1 | 1 |
| g-Hydroxy Butryic acid | 1 | 0 | 0 | 1 | 1 | 0 | 0 | 1 | 1 | 1 |
| N-Acetyl-L-Glutamic acid | 1 | 1 | 0 | 1 | 1 | 0 | 0 | 0 | 1 | 1 |
| Glycolic Acid | 1 | 0 | 0 | 0 | 1 | 0 | 0 | 1 | 1 | 1 |
| M-Tartaric Acid | 0 | 0 | 0 | 0 | 1 | 1 | 1 | 1 | 1 | 0 |
| Citraconic acid | 0 | 0 | 0 | 0 | 0 | 1 | 1 | 1 | 1 | 1 |
| Citric Acid | 1 | 1 | 1 | 1 | 0 | 0 | 0 | 0 | 0 | 0 |
| D-Galactonic Acid -γ -Lactone | 0 | 0 | 0 | 1 | 1 | 0 | 1 | 1 | 0 | 0 |
| D-Tartaric acid | 0 | 0 | 0 | 0 | 0 | 1 | 1 | 0 | 1 | 1 |
| Sorbic acid | 1 | 1 | 0 | 0 | 0 | 0 | 0 | 0 | 1 | 1 |
| N-Acetyl-Neuraminic acid | 0 | 1 | 1 | 0 | 0 | 0 | 0 | 0 | 1 | 1 |
| 4-Hydroxy Benzoic acid | 1 | 1 | 1 | 1 | 0 | 0 | 0 | 0 | 0 | 0 |
| D-Galacturonic acid | 1 | 1 | 1 | 0 | 0 | 0 | 0 | 0 | 0 | 0 |
| D-Saccharic Acid | 1 | 1 | 0 | 1 | 0 | 0 | 0 | 0 | 0 | 0 |
| Capric Acid | 0 | 0 | 0 | 0 | 1 | 0 | 0 | 0 | 1 | 1 |
| L-Galactonic Acid-γ -Lactone | 1 | 0 | 0 | 0 | 0 | 0 | 1 | 0 | 0 | 0 |
| Mucic acid | 0 | 0 | 0 | 0 | 1 | 0 | 0 | 1 | 0 | 0 |
| Tricarballylic Acid | 0 | 0 | 0 | 1 | 0 | 0 | 0 | 0 | 0 | 0 |
| *α-Hydroxy Glutaric Acid- γ-Lactone* | 1 | 0 | 0 | 0 | 0 | 0 | 0 | 0 | 0 | 0 |
| *a-Keto Valeric acid* | 1 | 0 | 0 | 0 | 0 | 0 | 0 | 0 | 0 | 0 |
| *Oxalic acid* | 1 | 0 | 0 | 0 | 0 | 0 | 0 | 0 | 0 | 0 |
| Itaconic acid | 0 | 0 | 0 | 0 | 0 | 0 | 0 | 0 | 0 | 0 |
| Sebacic acid | 0 | 0 | 0 | 0 | 0 | 0 | 0 | 0 | 0 | 0 |
| 2-Hydroxy Benzoic acid | 0 | 0 | 0 | 0 | 0 | 0 | 0 | 0 | 0 | 0 |
| **Polymers** |  |  |  |  |  |  |  |  |  |  |
| 3-0-β-D-Galactopyranosyl-D-Arabinose | 1 | 1 | 1 | 1 | 1 | 1 | 1 | 1 | 1 | 1 |
| Glycogen | 1 | 1 | 1 | 1 | 0 | 1 | 1 | 1 | 1 | 1 |
| α-Cyclodextrin | 1 | 1 | 1 | 0 | 1 | 1 | 1 | 1 | 1 | 1 |
| Tween 40 | 1 | 0 | 1 | 0 | 1 | 1 | 1 | 1 | 1 | 1 |
| β-Cyclodextrin | 1 | 1 | 1 | 0 | 0 | 1 | 1 | 1 | 1 | 1 |
| g-Cyclodextrin | 1 | 1 | 1 | 0 | 0 | 1 | 1 | 1 | 1 | 1 |
| Dextrin | 1 | 1 | 1 | 1 | 1 | 0 | 1 | 0 | 0 | 0 |
| Tween 20 | 1 | 0 | 1 | 1 | 0 | 0 | 0 | 0 | 1 | 0 |
| Tween 80 | 1 | 0 | 1 | 0 | 0 | 0 | 0 | 0 | 1 | 1 |
| Pectin | 1 | 1 | 0 | 0 | 1 | 0 | 0 | 0 | 0 | 0 |
| Inulin | 0 | 1 | 0 | 1 | 0 | 0 | 1 | 0 | 0 | 0 |
| Laminarin | 1 | 1 | 0 | 0 | 0 | 1 | 0 | 0 | 0 | 0 |
| *Chondroitin Sulphate C* | 0 | 0 | 1 | 0 | 0 | 0 | 0 | 0 | 0 | 0 |
| **Miscellaneous** |  |  |  |  |  |  |  |  |  |  |
| Glycerol | 1 | 1 | 1 | 1 | 1 | 1 | 1 | 1 | 0 | 1 |
| Inosine | 1 | 1 | 1 | 1 | 1 | 1 | 1 | 1 | 0 | 1 |
| Thymidine | 0 | 1 | 1 | 1 | 1 | 0 | 1 | 1 | 1 | 1 |
| Amygdalin | 1 | 1 | 1 | 1 | 0 | 1 | 0 | 1 | 1 | 1 |
| 2-Deoxy Adenosine | 1 | 1 | 1 | 1 | 0 | 1 | 1 | 0 | 1 | 0 |
| Adenosine | 1 | 1 | 1 | 1 | 0 | 1 | 1 | 0 | 0 | 1 |
| D-Lactic Acid Methyl Ester | 1 | 0 | 1 | 0 | 1 | 1 | 1 | 1 | 0 | 0 |
| Uridine | 1 | 1 | 1 | 1 | 0 | 0 | 0 | 0 | 0 | 1 |
| Salicin | 1 | 1 | 1 | 0 | 0 | 1 | 0 | 1 | 0 | 0 |
| Gelatin | 1 | 1 | 1 | 1 | 0 | 1 | 0 | 0 | 0 | 0 |
| 2,3-Butanediol | 0 | 0 | 0 | 1 | 0 | 0 | 0 | 0 | 1 | 1 |
| D-Ribono-1,4-Lactone | 1 | 0 | 1 | 1 | 0 | 0 | 0 | 0 | 0 | 0 |
| D-Fructose-6-Phosphate | 0 | 0 | 0 | 0 | 0 | 0 | 0 | 0 | 1 | 1 |
| Mono methyl succinate | 1 | 0 | 0 | 0 | 0 | 0 | 0 | 0 | 0 | 1 |
| 3-hydroxy 2-Butanone | 1 | 0 | 0 | 1 | 0 | 0 | 0 | 0 | 0 | 0 |
| N-Acetyl-D-Glucosaminitol | 0 | 0 | 0 | 1 | 0 | 0 | 0 | 1 | 0 | 0 |
| *D,L-α-Glycerol -phospahte* | 1 | 0 | 0 | 0 | 0 | 0 | 0 | 0 | 0 | 0 |
| D-Glucose-6-Phosphate | 0 | 0 | 0 | 0 | 0 | 0 | 0 | 0 | 1 | 0 |
| *D-Glucose-1-Phosphate* | 1 | 0 | 0 | 0 | 0 | 0 | 0 | 0 | 0 | 0 |
| 1,2-Propanediol | 0 | 0 | 0 | 0 | 0 | 0 | 0 | 0 | 0 | 0 |
| 2,3-Butanone | 0 | 0 | 0 | 0 | 0 | 0 | 0 | 0 | 0 | 0 |
| Sum | 140 | 123 | 127 | 121 | 100 | 98 | 103 | 105 | 109 | 109 |
