# Supplementary material for: Genomic and phenotypic insights into the ecology of Arthrobacter from Antarctic soils
Source: BMC Genomics. 2015 Feb 5;16(1):36. doi: 10.1186/s12864-015-1220-2 (PMC4326396; doi:10.1186/s12864-015-1220-2)
Supplement: Additional file 10: — Salinity tolerance profiles of three temperate Arthrobacter spp. and seven Antarctic Arthrobacter isolates determined by the BIOLOG PM9 Osmolyte plate. [file 12864_2015_1220_MOESM10_ESM.docx]

Additional file 10: Salinity tolerance profiles of three temperate *Arthrobacter* spp. and seven Antarctic *Arthrobacter* isolates determined by the BIOLOG PM9 Osmolyte plate.

|  | *A*.*chlorophenolicus* | *A*.*phenanthrenivorans* | *A*. sp. FB24 | I3 | H14 | H5 | H20 | 35/47 | Br18 | H41 |
| --- | --- | --- | --- | --- | --- | --- | --- | --- | --- | --- |
| 1% NaCl | 1 | 1 | 1 | 1 | 1 | 1 | 1 | 1 | 1 | 1 |
| 6% NaCl | 1 | 1 | 1 | 1 | 1 | 1 | 1 | 1 | 1 | 1 |
| NaCl 6% + KCl | 1 | 1 | 1 | 1 | 1 | 1 | 1 | 1 | 0 | 1 |
| 3% KCl | 1 | 1 | 1 | 1 | 1 | 1 | 1 | 1 | 1 | 1 |
| 1% Na formate | 1 | 1 | 1 | 1 | 1 | 0 | 1 | 1 | 1 | 1 |
| 1% Na lactate | 1 | 1 | 1 | 1 | 0 | 0 | 1 | 0 | 0 | 0 |
| Na Phosphate pH 7 20 mM | 1 | 1 | 1 | 1 | 1 | 1 | 1 | 1 | 1 | 1 |
| Na nitrate 10mM | 1 | 1 | 1 | 1 | 1 | 1 | 1 | 1 | 1 | 1 |
| 2% NaCl | 1 | 1 | 1 | 1 | 1 | 1 | 1 | 1 | 1 | 1 |
| 6% NaCl + Betaine | 1 | 1 | 1 | 1 | 1 | 1 | 1 | 1 | 1 | 1 |
| NaCl 6%+ L-proline | 1 | 1 | 1 | 1 | 1 | 1 | 1 | 1 | 1 | 1 |
| 4% KCl | 1 | 1 | 1 | 1 | 1 | 1 | 1 | 1 | 1 | 1 |
| 2% Na formate | 1 | 1 | 1 | 1 | 1 | 0 | 0 | 0 | 0 | 1 |
| 2% Na Lactate | 1 | 0 | 1 | 1 | 0 | 0 | 0 | 0 | 0 | 0 |
| Na Phosphate pH 7 50 mM | 1 | 1 | 1 | 1 | 1 | 1 | 1 | 1 | 1 | 1 |
| Na nitrate 20mM | 1 | 1 | 1 | 1 | 1 | 1 | 1 | 1 | 1 | 1 |
| 3% NaCl | 1 | 1 | 1 | 1 | 1 | 1 | 1 | 1 | 1 | 1 |
| 6% NaCl + N,N-Dimethyl Glycine | 1 | 1 | 1 | 1 | 1 | 1 | 1 | 1 | 1 | 1 |
| 6% NaCl + N-Acetyl L-Glutamine | 1 | 1 | 1 | 1 | 1 | 1 | 1 | 1 | 1 | 1 |
| 5% KCl | 1 | 1 | 1 | 1 | 1 | 1 | 1 | 1 | 0 | 1 |
| 3% Na formate | 1 | 1 | 1 | 0 | 0 | 0 | 0 | 0 | 0 | 0 |
| 3% Na lactate | 1 | 0 | 1 | 0 | 0 | 0 | 0 | 0 | 0 | 0 |
| Na Phosphate pH 7 100 mM | 1 | 1 | 1 | 1 | 1 | 1 | 1 | 1 | 1 | 1 |
| Na nitrate 40 mM | 1 | 1 | 1 | 1 | 1 | 1 | 1 | 1 | 1 | 1 |
| 4% NaCl | 1 | 1 | 1 | 1 | 1 | 1 | 1 | 1 | 1 | 1 |
| 6% NaCl + Sarcosine | 1 | 1 | 1 | 1 | 1 | 1 | 1 | 1 | 1 | 1 |
| 6% NaCl + β-Glutamic Acid | 1 | 1 | 1 | 1 | 1 | 1 | 1 | 1 | 0 | 0 |
| 6% KCl | 1 | 1 | 1 | 1 | 1 | 1 | 1 | 1 | 0 | 1 |
| 4% Na formate | 1 | 1 | 1 | 0 | 0 | 0 | 0 | 0 | 0 | 1 |
| 4% Na lactate | 1 | 0 | 1 | 0 | 0 | 0 | 0 | 0 | 0 | 0 |
| Na Phosphate pH 7 200 mM | 1 | 1 | 1 | 1 | 1 | 1 | 1 | 1 | 1 | 1 |
| Na nitrate 60 mM | 1 | 1 | 1 | 1 | 1 | 1 | 1 | 1 | 1 | 1 |
| 5% NaCl | 1 | 1 | 1 | 1 | 1 | 1 | 1 | 1 | 1 | 1 |
| 6% Nacl + Dimethyl sulphonyl propionate | 1 | 1 | 1 | 1 | 1 | 1 | 1 | 1 | 0 | 1 |
| 6% NaCl + γ–Amino-N-Butryic acid | 1 | 1 | 1 | 1 | 1 | 1 | 1 | 1 | 1 | 1 |
| 2% Na sulphate | 1 | 1 | 1 | 1 | 1 | 1 | 1 | 1 | 1 | 1 |
| 5% Na formate | 1 | 1 | 1 | 0 | 0 | 0 | 0 | 0 | 0 | 0 |
| 5% Na lactate | 1 | 0 | 1 | 0 | 0 | 0 | 0 | 0 | 0 | 0 |
| Na Benzoate pH 5.2 20 mM | 1 | 1 | 1 | 1 | 1 | 0 | 0 | 1 | 0 | 1 |
| Na nitrate 80 mM | 1 | 1 | 1 | 1 | 1 | 1 | 1 | 1 | 1 | 1 |
| 5.5% NaCl | 1 | 1 | 1 | 1 | 1 | 1 | 1 | 1 | 1 | 1 |
| 6% NaCl + MOPS | 1 | 1 | 1 | 1 | 1 | 1 | 1 | 1 | 1 | 1 |
| 6% NaCl + Glutathione | 1 | 1 | 1 | 1 | 1 | 1 | 1 | 1 | 1 | 1 |
| 3% Na sulphate | 1 | 1 | 1 | 1 | 1 | 1 | 1 | 1 | 1 | 1 |
| 6% Na formate | 1 | 1 | 1 | 0 | 0 | 0 | 0 | 0 | 0 | 0 |
| 6% Na lactate | 1 | 0 | 1 | 0 | 0 | 0 | 0 | 0 | 0 | 0 |
| Na Benzoate pH 5.2 50 mM | 1 | 1 | 1 | 1 | 0 | 0 | 0 | 0 | 0 | 0 |
| Na nitrate 100 mM | 1 | 1 | 1 | 1 | 1 | 1 | 1 | 1 | 1 | 1 |
| 6% NaCl | 1 | 1 | 1 | 1 | 1 | 1 | 1 | 1 | 1 | 1 |
| 6% NaCl + Ectoine | 1 | 1 | 1 | 1 | 1 | 1 | 1 | 1 | 1 | 1 |
| 6% NaCl + glycerol | 1 | 1 | 1 | 1 | 1 | 1 | 1 | 1 | 1 | 1 |
| 4% Na sulphate | 1 | 1 | 1 | 1 | 1 | 1 | 1 | 1 | 1 | 1 |
| 2% urea | 1 | 1 | 1 | 1 | 1 | 1 | 1 | 1 | 1 | 1 |
| 7% Na lactate | 1 | 0 | 1 | 0 | 0 | 0 | 0 | 0 | 0 | 0 |
| Na Benzoate pH 5.2 100 mM | 1 | 1 | 1 | 1 | 0 | 0 | 0 | 0 | 0 | 0 |
| Na nitrite 10 mM | 1 | 1 | 1 | 1 | 1 | 1 | 1 | 1 | 1 | 1 |
| 6.5% NaCl | 1 | 1 | 1 | 1 | 1 | 1 | 1 | 1 | 1 | 1 |
| 6% NaCl + Choliine | 1 | 1 | 1 | 1 | 1 | 1 | 1 | 1 | 1 | 1 |
| 6% NaCl + trehalose | 1 | 1 | 1 | 1 | 1 | 1 | 1 | 1 | 1 | 1 |
| 5% Na sulphate | 1 | 1 | 1 | 1 | 1 | 1 | 1 | 1 | 1 | 1 |
| 3% urea | 1 | 1 | 1 | 1 | 1 | 1 | 0 | 1 | 1 | 1 |
| 8% Na lactate | 1 | 0 | 1 | 0 | 0 | 0 | 0 | 0 | 0 | 0 |
| Na Benzoate pH 5.2 200 mM | 1 | 1 | 1 | 0 | 1 | 0 | 0 | 0 | 0 | 0 |
| Na nitrite 20 mM | 1 | 1 | 1 | 1 | 1 | 1 | 1 | 1 | 1 | 1 |
| 7% NaCl | 1 | 1 | 1 | 1 | 1 | 1 | 1 | 1 | 1 | 1 |
| 6% NaCl + Phosphoryl choline | 1 | 1 | 1 | 1 | 1 | 1 | 1 | 1 | 1 | 1 |
| 6% NaCl + Trimethylamine-N-oxide | 1 | 1 | 1 | 1 | 1 | 1 | 1 | 1 | 1 | 1 |
| 5% ethylene glycol | 1 | 1 | 1 | 1 | 1 | 1 | 1 | 1 | 1 | 1 |
| 4% urea | 1 | 1 | 1 | 1 | 1 | 1 | 0 | 1 | 1 | 0 |
| 9% Na lactate | 1 | 0 | 1 | 0 | 0 | 0 | 0 | 0 | 0 | 0 |
| Ammonium sulphate pH8 10 mM | 1 | 1 | 1 | 1 | 1 | 1 | 1 | 1 | 1 | 1 |
| Na nitrite 40 mM | 1 | 1 | 1 | 1 | 1 | 1 | 1 | 1 | 0 | 1 |
| 8% NaCl | 1 | 1 | 1 | 0 | 1 | 0 | 1 | 1 | 1 | 0 |
| 6% NaCl + creatinine | 1 | 1 | 1 | 1 | 1 | 1 | 1 | 1 | 1 | 1 |
| 6% NaCl + Trimethylamine-N-oxide | 1 | 1 | 1 | 1 | 1 | 1 | 1 | 1 | 1 | 1 |
| 10% ethylene glycol | 1 | 1 | 1 | 1 | 1 | 1 | 1 | 1 | 1 | 1 |
| 5% urea | 1 | 1 | 1 | 1 | 1 | 0 | 0 | 1 | 0 | 0 |
| 10% Na lactate | 0 | 0 | 0 | 0 | 0 | 0 | 0 | 0 | 0 | 0 |
| Ammonium sulphate pH8 20 mM | 1 | 1 | 1 | 1 | 1 | 1 | 1 | 1 | 1 | 1 |
| Na nitrite 60 mM | 1 | 1 | 1 | 1 | 1 | 0 | 1 | 1 | 0 | 1 |
| 9% NaCl | 1 | 1 | 1 | 0 | 1 | 0 | 1 | 1 | 0 | 0 |
| 6% NaCl + creatinine | 1 | 1 | 1 | 1 | 1 | 1 | 1 | 1 | 1 | 1 |
| 6% NaCl + Octopine | 1 | 1 | 1 | 1 | 1 | 1 | 1 | 1 | 1 | 1 |
| 15% ethylene glycol | 1 | 1 | 1 | 1 | 1 | 1 | 1 | 1 | 1 | 1 |
| 6% urea | 1 | 1 | 1 | 0 | 0 | 0 | 0 | 1 | 0 | 0 |
| 11% Na lactate | 0 | 0 | 0 | 0 | 0 | 0 | 0 | 0 | 0 | 0 |
| Ammonium sulphate pH8 50 mM | 1 | 1 | 1 | 1 | 1 | 1 | 1 | 1 | 1 | 1 |
| Na nitrite 80 mM | 1 | 1 | 1 | 1 | 1 | 0 | 1 | 1 | 0 | 1 |
| 10% NaCl | 1 | 1 | 1 | 0 | 1 | 0 | 1 | 1 | 0 | 0 |
| 6% Nacl + L-Carnitine | 1 | 1 | 1 | 1 | 1 | 1 | 1 | 1 | 1 | 1 |
| 6% NaCl + Trigonelline | 1 | 1 | 1 | 1 | 1 | 1 | 1 | 1 | 1 | 1 |
| 20% ethylene glycol | 1 | 1 | 1 | 1 | 1 | 1 | 1 | 1 | 1 | 1 |
| 7% urea | 1 | 1 | 1 | 0 | 0 | 0 | 0 | 0 | 0 | 0 |
| 12% Na lactate | 0 | 0 | 0 | 0 | 0 | 0 | 0 | 0 | 0 | 0 |
| Ammonium sulphate pH8 100 mM | 1 | 1 | 1 | 1 | 1 | 1 | 1 | 1 | 1 | 1 |
| Na nitrite 100 mM | 1 | 1 | 1 | 1 | 1 | 0 | 1 | 0 | 0 | 0 |
| **Sum** | 93 | 85 | 93 | 76 | 76 | 65 | 71 | 74 | 61 | 69 |
