# Supplementary material for: Genomic and phenotypic insights into the ecology of Arthrobacter from Antarctic soils
Source: BMC Genomics. 2015 Feb 5;16(1):36. doi: 10.1186/s12864-015-1220-2 (PMC4326396; doi:10.1186/s12864-015-1220-2)
Supplement: Additional file 11: — A description of taxon-specific primers used for qPCR assays. [file 12864_2015_1220_MOESM11_ESM.pptx]

## Slide 1
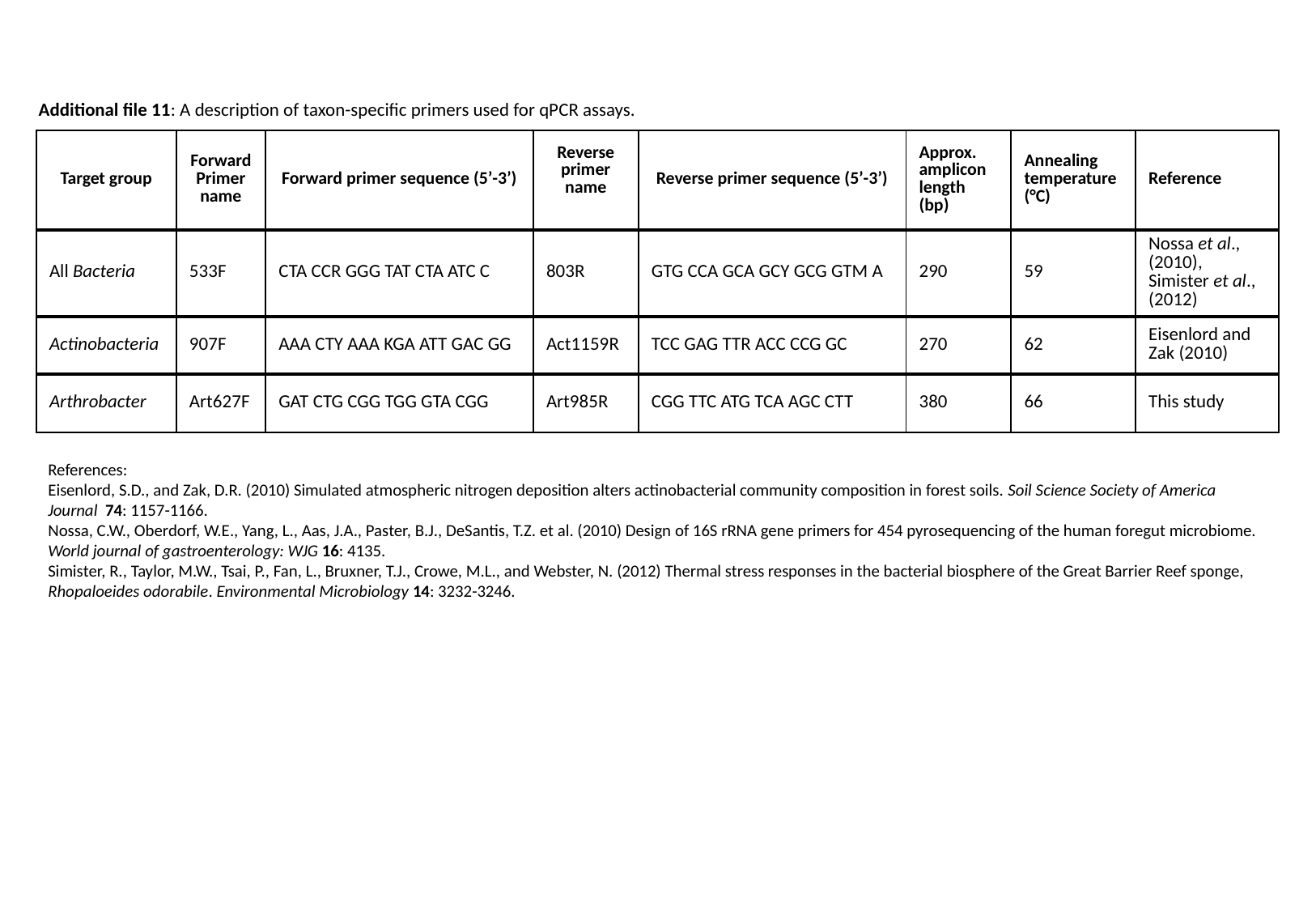

Additional file 11: A description of taxon-specific primers used for qPCR assays.
| Target group | Forward Primer name | Forward primer sequence (5’-3’) | Reverse primer name | Reverse primer sequence (5’-3’) | Approx. amplicon length (bp) | Annealing temperature (°C) | Reference |
| --- | --- | --- | --- | --- | --- | --- | --- |
| All Bacteria | 533F | CTA CCR GGG TAT CTA ATC C | 803R | GTG CCA GCA GCY GCG GTM A | 290 | 59 | Nossa et al., (2010), Simister et al., (2012) |
| Actinobacteria | 907F | AAA CTY AAA KGA ATT GAC GG | Act1159R | TCC GAG TTR ACC CCG GC | 270 | 62 | Eisenlord and Zak (2010) |
| Arthrobacter | Art627F | GAT CTG CGG TGG GTA CGG | Art985R | CGG TTC ATG TCA AGC CTT | 380 | 66 | This study |
References:
Eisenlord, S.D., and Zak, D.R. (2010) Simulated atmospheric nitrogen deposition alters actinobacterial community composition in forest soils. Soil Science Society of America Journal 74: 1157-1166.
Nossa, C.W., Oberdorf, W.E., Yang, L., Aas, J.A., Paster, B.J., DeSantis, T.Z. et al. (2010) Design of 16S rRNA gene primers for 454 pyrosequencing of the human foregut microbiome. World journal of gastroenterology: WJG 16: 4135.
Simister, R., Taylor, M.W., Tsai, P., Fan, L., Bruxner, T.J., Crowe, M.L., and Webster, N. (2012) Thermal stress responses in the bacterial biosphere of the Great Barrier Reef sponge, Rhopaloeides odorabile. Environmental Microbiology 14: 3232-3246.
